# Tubulin alpha-1b chain was identified as a prognosis and immune biomarker in pan-cancer combing with experimental validation in breast cancer

**DOI:** 10.1038/s41598-024-58982-z

**Published:** 2024-04-08

**Authors:** Yiyang Wang, Yongxiang Li, Yubo Jing, Yuqi Yang, Haiyan Wang, Dilimulati Ismtula, Chenming Guo

**Affiliations:** 1 Department of Breast Surgery, Center of Digestive and Vascular, The First Affiliated Hospital of Xinjiang Medical University, Urumqi, 830054 China; 2 The First Clinical Medical College of Xinjiang Medical University, Urumqi, 830054 China

**Keywords:** Pan-cancer, TUBA1B, Prognosis, Tumor immunity, Methylation, Cancer, Breast cancer, Biomarkers

## Abstract

The α-tubulin subtype, Tubulin α-1b chain (TUBA1B), has been shown to influence immune cell infiltration, cancer growth, and survival across various malignancies. However, a comprehensive study has not yet been undertaken examining the immunological and predictive effects of TUBA1B in a pan-carcinoma context. Using data from TCGA, GEO, and other databases, we analyzed TUBA1B expression across various carcinoma types using transcriptional profiling, prognostic implications, genetic and epigenetic alterations, methylation patterns, and immunological significance. To validate our findings, we conducted Western blot analysis to assess TUBA1B protein levels in matched breast cancer tissue samples and performed CCK-8 proliferation assay, flow cytometry, transwell invasion, and migration assays to comprehensively examine the functional impact of TUBA1B on breast cancer cells. Our pan-cancer analysis found TUBA1B upregulation across most tumor types, with varying expression patterns in distinct immune and molecular subtypes. High TUBA1B expression was an independent risk factor and associated with poor prognoses in several cancers, including BRCA, KICH, LGG, LUAD, and MESO. TUBA1B also demonstrates moderate to high diagnostic accuracy in most tumor types. Increased m6A methylation levels were observed in the TUBA1B gene, while its promoter region displayed low methylation levels. TUBA1B's expression impacted some cancers by elevating tumor mutation burden, microsatellite instability, neoantigen formation, immune cell infiltration, and the modulation of immune checkpoints. Functional enrichment analysis highlights TUBA1B’s involvement in important cellular processes such as the cell cycle, p53 signaling, cell senescence, programmed cell death, and the regulation of immune-related pathways. Moreover, our study reveals higher TUBA1B protein expression in breast cancer tissues compared to adjacent tissues. In vitro experiments confirm that TUBA1B deletion reduces breast cancer cell proliferation, invasion, and migration while increasing apoptosis. In conclusion, our study suggests that TUBA1B could potentially serve as a diagnostic marker for predicting cancer immunological profiles and survival outcomes and shed light on the expression and role of TUBA1B in breast cancer, providing a solid foundation for considering it as a promising therapeutic target for breast cancer patient treatment.

## Introduction

Cancer, the leading cause of global mortality, significantly affects human life due to its rapidly increasing incidence and associated high mortality rates^1^. Despite developments in multiple antitumor treatment modalities, such as surgery, radio-frequency excision, chemotherapy, immunological therapy, and targeted treatment^2^, a comprehensive cure for cancer remains elusive. Thus, there is an urgent need for pan-cancer investigations focused on specific genes and their relevance across diverse malignancies to provide valuable insights and innovative strategies for cancer treatment.

Since the discovery of microtubules over 50 years ago, their functionality remains under continuous investigation, holding significant importance for studying human diseases^3^. Microtubules are slender cytoskeleton filaments measuring 25 nm in diameter, composed of heterodimers comprising spherical α-tubulin and β-tubulin molecules. They are pivotal in cell division, structural integrity, cellular movement, and intracellular transport^4^. Recent research has demonstrated that the regulation of tubulin's posttranslational modifications (PTMs) can impact various cellular processes, including mitosis, cell cycle, cardiomyocyte contraction, neuronal function, and consequently, is implicated in neurodegenerative diseases, heart conditions, bleeding disorders, and a range of cancers^5–7^.

Tubulin α-1b chain (TUBA1B) is an important α-tubulin isoform with significant implications. For instance, it has been observed to impact immune cell infiltration within the tumor microenvironment of liver hepatocellular carcinoma (LIHC) and influence the responsiveness to immunotherapy, thereby significantly affecting their prognosis^8,9^, potentially attributed to TUBA1B overexpression, which has been linked to paclitaxel resistance in LIHC patients^10^. Additionally, TUBA1B has received attention for its upregulation in nephroblastoma^11^ and its characterization as a crucial regulator in osteosarcoma^12^. In addition, low TUBA1B expression has been associated with adverse effects on the overall survival (OS) of patients with colon adenocarcinoma (COAD), leading to CD8 + T cell depletion and impacting COAD patients' response to immunotherapy^13^. Furthermore, it serves as a significant signature in LIHC, pancreatic adenocarcinoma (PAAD), and glioblastoma multiforme (GBM) for constructing cancer classification models and predicting patient outcomes^14–16^. In summary, the TUBA1B gene shows promise as a biomarker for predicting cancer treatment outcomes and serves as a predictor of immunotherapy responses. Nevertheless, a comprehensive pan-carcinoma investigation into TUBA1B, aimed at elucidating its biological role and mechanisms of action, is currently lacking.

In this study, we conducted a comprehensive pan-carcinoma investigation of TUBA1B to explore its potential as a novel immunotherapy strategy for tumor treatment by assessing its structural characteristics and functions.

## Material and methods

### Data and differential analysis of TUBA1B expression in pan-cancer

We collected and analyzed data from the UCSC XENA platform (https://xenabrowser.net/datapages/), which comprised 15,776 samples based on 33 cancer-related RNA-sequencing expression profiles with clinicopathological information and survival data of relevant patients. Expression spectrum data formatted in Transcripts Per Million (TPM) was transformed by log2 and merged with subsequent analyses. Data collection and cleaning were performed using the “tidyverse” and “reshape2” packages, while data analysis and visualization were conducted with the “rstatix,” “car,” and “ggplot2” packages within the R software (Version 4.0.3, https://www.r-project.org/). To further corroborate TUBA1B expression levels, we retrieved gene expression profiles from 8 Gene Expression Omnibus (GEO) datasets using the “GEOquery” package, including GSE42568 (platform: GPL570), GSE66791 (platform: GPL570), GSE20916 (platform: GPL570), GSE66791 (platform: GPL570), GSE105261 (platform: GPL10558), GSE121248 (platform: GPL570), GSE15471 (platform: GPL570) and GSE54129 (platform: GPL570). Afterward, the data were normalized using the “normalizeBetweenArrays” function within the “limma” package.

Additionally, we utilized the Tumor Immune System Interaction Database (TISIDB) (http://cis.hku.hk/TISIDB/index.php)^17^, a web-based analytical platform, to investigate the relationship between TUBA1B expression, immunological subtypes, and pan-cancer molecular subtypes.

To validate differential TUBA1B expression at the protein level, we obtained immunohistochemical images from the Human Protein Atlas (HPA) database (https://www.proteinatlas.org/)^18^, which features 16 cancer tissues and their corresponding healthy tissues, exhibiting varying TUBA1B expressions and protein content.

### Correlation analysis of TUBA1B expression level and prognosis and diagnostic potential of pan-cancer

We investigated the relationship between TUBA1B expression and cancer patient prognostic indicators, including overall survival (OS), disease-specific survival (DSS), and progression-free survival (PFS) using survival data from the TCGA database for each sample. COX regression and Kaplan–Meier (KM) curve plotting were performed using the “survival” and “survminer” packages. Furthermore, we employed the “ggplot2” package to generate forest and Venn plots for visualizing the results. In addition to the TCGA database, we assessed the association between TUBA1B expression and patient survival outcomes using the PrognoScan database (http://dna00.bio.kyutech.ac.jp/PrognoScan/index.html)^19^, incorporating 15 datasets based on 8 different tumor types for our analysis.

The R “pROC” package was used to analyze ROC curves to evaluate TUBA1B's predictive value in distinguishing between TCGA tumor tissues and corresponding GTEx and TCGA normal tissues. The area under the curve (AUC) serves as a diagnostic performance indicator, with values closer to 1 indicating higher diagnostic accuracy. AUC values between 0.7 and 0.9 denote moderate diagnostic capacity for TUBA1B, while AUC values exceeding 0.9 signify strong diagnostic ability.

### Build and calibrate nomograms

To evaluate the factors influencing patient outcomes, we performed both univariate and multivariate Cox regression analyses. Factors with *p* < 0.1 in the univariate analysis were included in the multivariate Cox analysis. We used the median value as a threshold to categorize TUBA1B expression groups, treating them as independent factors. The parameters selected for the multivariate Cox regression analysis were integrated into a nomogram, and the prediction accuracy of the nomogram was determined using the consistency index (C-index), with 1000 replicates. Additionally, calibration curves were plotted to assess the agreement between predicted and actual survival outcomes.

### Use of online databases

We accessed the cBioPortal database (https://www.cbioportal.org)^20^ and selected the “TCGA Pan-Cancer Atlas Studies” dataset, which comprises 32 studies and a total of 10,967 samples. In this dataset, we investigated the genetic alterations of TUBA1B across various cancer types by analyzing 10,443 mutant data samples through the “OncoPrint” module. Additionally, we utilized the “Cancer Types Summary” module to assess the frequency of TUBA1B alterations, the number of gene mutations, mutation types, and copy number variants (CNV) for each specific malignancy. To identify the precise mutation sites within TUBA1B, we used the “Mutations” module, examined the protein’s 3D structure, and explored associated mutations, providing a more comprehensive insight into its genetic alterations.

The Gene Set Cancer Analysis (GSCA) database^21^ (http://bioinfo.life.hust.edu.cn/GSCA/#/) was used to assess the copy number variation (CNV%) within each cancer type. We also explored the relationships between TUBA1B expression and CNV, as well as the impact of CNV in TUBA1B on the prognosis of cancer patients. Additionally, we analyzed the association between TUBA1B methylation levels and cancer patient prognosis.

To obtain information about TUBA1B's presence within various CNV and Single Nucleotide Variation (SNV) types across pan-cancer, we referred to The Catalogue of Somatic Mutations in Cancer (COSMIC) (https://cancer.sanger.ac.uk/cosmic). Furthermore, we used the UALCAN database (http://ualcan.path.uab.edu/index.html)^22^ to compare TUBA1B promoter methylation levels between normal tissues and TCGA samples in different malignancies. Methylation levels were represented by β values, ranging from 0 (unmethylated) to 1 (completely methylated). Distinct β cutoffs were employed to indicate high (0.7–0.5) or low methylation (0.3–0.25).

### Correlation analysis of TUBA1B expression with immunity

First, we analyzed the relationship between TUBA1B and key immunological parameters, including microsatellite instability (MSI), tumor mutational burden (TMB), and neoantigen (NEO) in various cancer types, utilizing data from the Sangerbox 3.0 online database (http://vip.sangerbox.com/). Then, to compute StromalScore, ImmuneScore, and ESTIMATEScore, we used the “GSVA” and “org.Hs.eg.db” packages following the ESTIMATE algorithm^23^. Subsequently, we explored the co-expression patterns of TUBA1B with immune checkpoint molecules. We obtained a list of immunoactivatory and immunosuppressive genes from the Gene Set Enrichment Analysis (GSEA) database (https://www.gsea-msigdb.org/gsea/msigdb/index.jsp). We further investigated the association between TUBA1B expression levels and the expression of these immune-related genes.

The single-sample GSEA (ssGSEA) algorithm^24^ was used to evaluate the infiltration levels of 24 immune cell types across pan-cancer. Next, we investigated the relationship between TUBA1B expression and the levels of tumor-associated immune cell infiltrates using the Timer2.0 database (http://timer.cistrome.org/)^25^. In our analysis within the “Immune” module, we utilized the EPIC, TIMER, CIBERSORT, and MCPCOUNTER algorithms to assess the presence of cancer-associated fibroblasts (CAFs), CD8 + T cells, CD4 + T cells, regulatory T cells (Tregs), B cells, macrophages, and natural killer cells (NKs). The correlation between TUBA1B expression and CAF abundance was visually represented using scatter plots.

### Biological significance of TUBA1B in pan-carcinoma

To investigate the proteins that interact with TUBA1B, we employed the STRING database (https://string-db.org/). We set specific parameters to retrieve experimentally determined TUBA1B binding proteins and constructed a protein–protein interaction (PPI) network. The first 100 genes related to TUBA1B, as well as their correlations with different genes, were validated using the “Similar Genes Detection” module in the Gene Expression Profiling Interactive Analysis (GEPIA2) database (http://gepia2.cancer-pku.cn/#index)^26^. By creating a Venn diagram, we identified genes shared between TUBA1B-binding proteins and related genes. Subsequently, we examined the correlation between TUBA1B and these shared genes using the Timer 2.0 database. Finally, we conducted a functional enrichment analysis of TUBA1B using the “clusterProfiler” and “org.Hs.eg.db” packages to gain insights into its biological functions.

Furthermore, we categorized TUBA1B expression into high and low groups using 50% as the threshold. Differential expression analysis of individual genes was conducted utilizing the “DESeq2” package. All genes with their corresponding “log2FoldChange” values were included in Gene Set Enrichment Analysis (GSEA). To elucidate functional disparities between the high and low expression groups in various cancer cohorts, the “c2.cp.v7.2.symbols.gmt” gene set from MSigDB (https://www.gsea-msigdb.org/gsea/index.jsp)^27^ was employed and repeated 10,000 times. The ridge diagram visually represents the top 10 “Reactom pathways” for each cancer type.

### Cell culture and transfection

Two human breast cancer cell lines, MDA-MB-231 CL-0150B and MDA-MB-468 CL-0290A, were obtained from Procell (Procell Life Science & Technology Co., Ltd., China) and authenticated by STR analysis. The cells were cultured in DMEM (PM150210, Procell Life Science & Technology Co., Ltd., China) at 37 °C with 5% CO_2_ and supplemented with 10% fetal bovine serum (FBS) (10,091,148, Gibco, China), 100 µg/mL streptomycin, and 100U/mL penicillin (SV30010, Hyclone, USA). Transfection of siRNA-NC and siRNA-TUBA1B into MDA-MB-231 and MDA-MB-468 cells was performed using the Lipofectamine™RNAiMAX transfection reagent (13,778,030, Invitrogen, Carlsbad CA USA), following the manufacturer’s protocol. All siRNA duplexes were purchased from Gemma (Suzhou, China) (Table S1).

### The RNA extraction and real-time fluorescent quantitative PCR

Total RNA was extracted using TRIZOL (15,596–018, Ambion, USA) and treated twice with phenol–chloroform to purify RNA further, followed by RQ1 DNase (M6101, Promega, Madison, WI, USA) to remove DNA. The extracted and purified RNA was reverse transcribed into cDNA using a reverse transcription cassette (R323-01, Vazyme, China) on a thermal cycler (T100, Bio-Rad, USA), and the detection of qPCR was performed on an ABI QuantStudio5 using GAPDH as an internal reference. The primer sequences used were as follows: GAPDH-F: GGTCGGAGTCAACGGATTTG; GAPDH-R: GGAAGATGGTGATGGGATTTC; TUBA1B-F: TGACCTGATGTATGCCAAG; TUBA1B-R: TTAGTATTCCTCTCCTTCTTCC. Data analysis was performed using the 2-ΔΔCT method, and statistical analysis was conducted using T-tests. GraphPad Prism software (Version 8.0, San Diego, CA) was used for data analysis and visualization.

### Patient tissue collection and Western blot

This study included seven breast cancer patients who underwent surgery at the Department of Breast Surgery, the First Affiliated Hospital of Xinjiang Medical University, between April 2022 and December 2022. None of the patients had received adjuvant radiotherapy, chemotherapy, or hormonal therapy before surgery. The mean age of the patients was 53 years, with an age range of 27–68 years. The clinical research protocol of this study was reviewed and approved by the Ethics Committee of the First Affiliated Hospital of Xinjiang Medical University (Lun Review No.). All patients provided written informed consent, and the study was conducted following the principles of the Declaration of Helsinki.

Tissues were lysed in ice-cold RIPA buffer (PR20001, Proteintech, China) supplemented with a protease inhibitor cocktail (4,693,116,001, Sigma, USA) and incubated on ice for 30 min. Subsequently, the samples were boiled in boiling water with protein loading buffer (P1040, Solarbio, China) for 10 min and loaded onto 10% SDS-PAGE gels. After electrophoresis, the proteins were transferred to 0.45 mm PVDF membranes (ISEQ00010, Millipore, USA). After cutting out the corresponding bands according to the molecular weight, the PVDF membranes were then blocked with milk for 1 h at room temperature, followed by overnight incubation with primary antibodies at 4 °C. Afterward, the membranes were incubated with secondary antibodies conjugated to horseradish peroxidase for 45 min at room temperature. The protein bands on the membrane were visualized using chemiluminescence with an enhanced ECL reagent (P0018FM, Beyotime, China). Image J software was used to analyze the gray value, and the gray value of TUBA1B protein/internal reference protein gray value was used to calculate the relative expression of TUBA1B protein.

### Cell proliferation and apoptosis assay

The CCK-8 assay (CCK-8, 40203ES76, Yeasen, Shanghai, China) was used for cell proliferation assay. The selected cells were seeded in 24-well culture plates. Both control and experimental groups were treated as indicated, and a vial without cells was used as a blank control. Following an incubation period at 37 °C with 5% CO_2_, CCK-8 solution was added, and the cells were further incubated for 3 h at 37 °C. Subsequently, the optical density of the cells was measured using a Microplate Reader (ELX800, Biotek, USA) at an absorbance of 450 nm. For apoptosis detection, we used the Annexin V-APC/7-ADD apoptosis detection kit (40304ES60, Yeasen, Shanghai, China). Briefly, the selected cells were seeded in 12-well plates, cultured for 24 h, and then transfected with the plasmid for 48 h. Treated and control cells were incubated with 5 μl Annexin V-APC and 10 μl PI reagent for 5 min at room temperature in the dark and 5 min, respectively. Apoptosis levels were subsequently measured using flow cytometry (FACSCanto, BD, USA).

### Cell invasion and migration assay

In vitro, cell invasion and migration assays were conducted using Transwell chambers (3422, Corning, USA). Transfected and control MDA-MB-231 and MDA-MB-468 cells were seeded in Transwell chambers, with or without Matrigel, featuring an 8 µm filter. The Transwell chamber was inserted with 600 μl of 10% FBS medium (10,091,148, Gibco, China) in the lower chamber to act as a chemoattractant. The chambers were then incubated for 24 h at 37 °C in 5% CO_2_. After incubation, any remaining cells on the upper membrane surface of the insert were removed using a cotton swab, then the total number of cells that had invaded the lower chamber was fixed with 4% paraformaldehyde (P0099, Beyotime, China) for 30 min and stained with 0.1% crystal violet (C0121, Beyotime, China). The invasive and migrating cells were observed and counted under an inverted microscope (MF52-N, Mshot, China) at 200 × magnification. The above cell experiments were repeated 3 times for each sample.

### Statistical analysis

The R software (vs. 4.0.3, https://www. R-project.org/) was used for statistical analysis, and the “ggplot2” package was used for data visualization. According to the median level of TUBA1B mRNA expression, samples were divided into high- and low-expression groups. The Mann–Whitney U test was utilized to examine variations in expression levels of TUBA1B in unmatched samples, and Wilcoxon signed rank test was employed for paired samples. The Spearman correlation coefficient was used to investigate the association between TUBA1B expression and m6A methylation regulators, TMB, MSI, NEO, immune score, and immune-related genes. *P* < 0.05 is considered statistically significant.

## Ethical approval

The studies involving humans were approved by the Ethics Committee of The First Affiliated Hospital of Xinjiang Medical University (No. 230714-08) and were conducted following local legislation and institutional requirements. The participants provided their written informed consent to participate in this study.

## Results

### Gene Expression Analysis of TUBA1B

**Figure 1:**
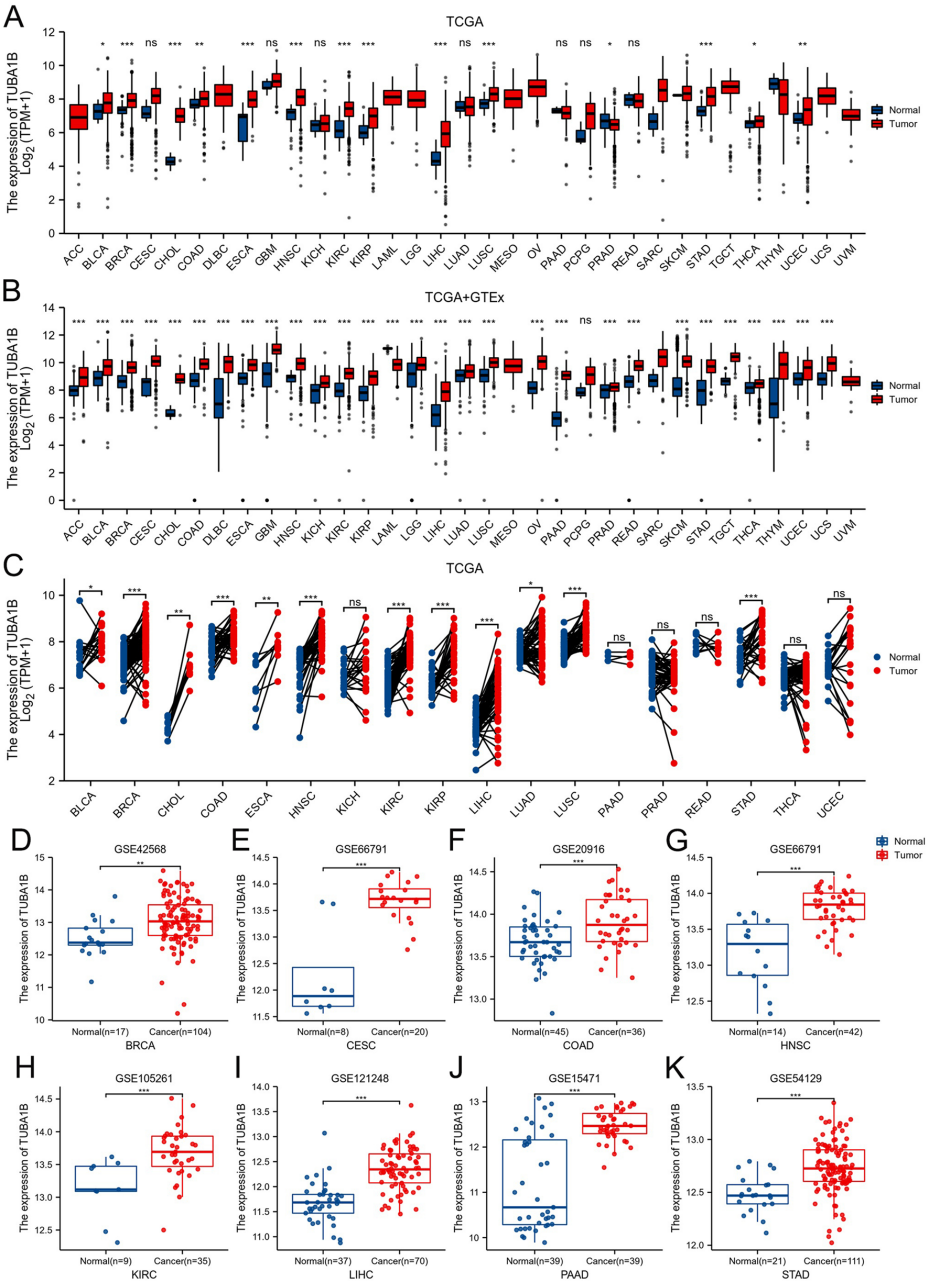
TUBA1B expression variations across 33 cancer types. (**A**) Comparison of TUBA1B mRNA expression between TCGA tumor samples and corresponding normal tissues. (**B**) Differences in TUBA1B mRNA expression between tumor and normal tissues, integrating data from TCGA and GTEx datasets. (**C**) TUBA1B mRNA expression in TCGA tumor samples compared to paired normal tissues. Analysis of TUBA1B expression differences was conducted using GEO datasets for specific cancers: (**D**) BRCA (GSE42568), (**E**) CESC (GSE66791), (**F**) COAD (GSE20916), (**G**) HNSC (GSE66791), (**H**) KIRC (GSE105261), (**I**) LIHC (GSE121248), (**J**) PAAD (GSE15471), and (**K**) STAD (GSE54129). * *p* < 0.05, ** *p* < 0.01, *** *p* < 0.001.

Figure 1A shows the TUBA1B expression levels across various cancer types, including bladder urothelial carcinoma (BLCA), breast invasive carcinoma (BRCA), cholangiocarcinoma (CHOL), COAD, esophageal carcinoma (ESCA), head and neck squamous cell carcinoma (HNSC), kidney renal clear cell carcinoma (KIRC), kidney renal papillary cell carcinoma (KIRP), LIHC, lung squamous cell carcinoma (LUSC), prostate adenocarcinoma (PRAD), stomach adenocarcinoma (STAD), thyroid carcinoma (THCA), and uterine corpus endometrial carcinoma (UCEC). Comparative analysis showed that TUBA1B mRNA expression levels were significantly elevated in each of these cancer types compared to the corresponding healthy tissue levels.

To determine the reliability of our findings, we matched GTEx normal tissue with TCGA cancer tissue, which revealed a significant upregulation in TUBA1B expression across 28 different cancer types, including adrenocortical carcinoma (ACC), BLCA, BRCA, cervical squamous cell carcinoma and endocervical adenocarcinoma (CESC), CHOL, COAD, diffuse large B-cell lymphoma (DLBC), ESCA, GBM, HNSC, kidney chromophobe (KICH), KIRC, KIRP, brain lower grade glioma (LGG), LIHC, lung adenocarcinoma (LUAD), LUSC, ovarian serous cystadenocarcinoma (OV), PAAD, PRAD, rectum adenocarcinoma (READ), skin cutaneous melanoma (SKCM), STAD, testicular germ cell tumors (TGCT), THCA, thymoma (THYM), UCEC and uterine carcinosarcoma (UCS). Comparatively, TUBA1B expression was down-regulated compared to healthy tissues only in LAML (*p* < 0.05; Fig. 1B).

In paired samples from 18 malignancies, we discovered that TUBA1B mRNA expression patterns were significantly elevated in BLCA, BRCA, CHOL, COAD, ESCA, HNSC, KIRC, KIRP, LIHC, LUAD, LUSC, and STAD compared to their matched normal tissues (*p* < 0.05; Fig. 1C). Additionally, we validated these findings in datasets from the GEO database, specifically in BRCA (*p* = 0.001), CESC (*p* = 3e-04), COAD (*p* = 5.3e-04), HNSC (*p* = 2.2e-04), KIRC (*p* = 7e-04), LIHC (*p* = 3.5e-12), PAAD (*p* = 1.4e-07) and STAD (*p* = 2.2e-05), demonstrating elevated levels compared to their corresponding matched tissues (Fig. 1D–K). Finally, we utilized the HPA database to assess the differential TUBA1B protein levels expression in normal and malignant tissues, including BLCA, BRCA, CESC, CHOL, COAD, KIRC, LIHC, LUAD, LUSC, OV, PAAD, PRAD, READ, SKCM, STAD and TGCT, and the protein-level findings were consistent with the previously observed mRNA expression values (Fig. S1).

**Figure 2:**
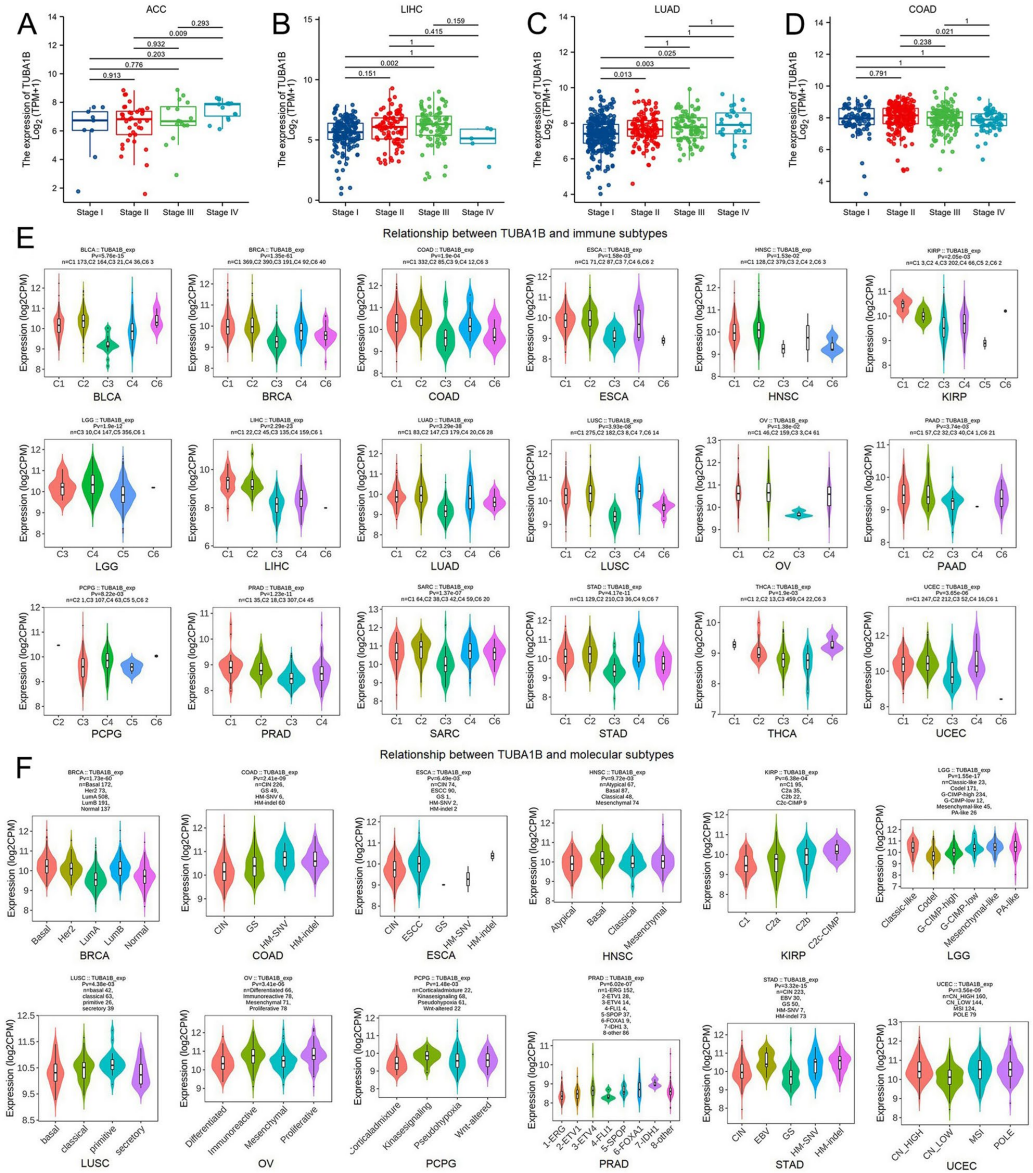
Correlation between TUBA1B expression and tumor stages in different cancers, including ACC (**A**), LIHC (**B**), LUAD (**C**), and COAD (**D**). (**E**) Relationships between immune subtypes and TUBA1B expression across various TCGA tumors, encompassing BLCA, BRCA, COAD, ESAD, HNSC, KIRP, LGG, LIHC, LUAD, LUSC, OV, PAAD, PCPG, PRAD, SARC, STAD, THCA, and UCEC. (**F**) Associations between molecular subtypes and TUBA1B expression in diverse TCGA tumors, comprising BRCA, COAD, ESCA, HNSC, KIRP, LGG, LUSC, OV, PCPG, PRAD, STAD, and UCEC.

Our investigation into the correlation between TUBA1B expression and tumor stages revealed that TUBA1B levels increased with advancing stages in ACC, LIHC, and LUAD, suggesting the potential of TUBA1B as a prognostic marker in these tumors (Fig. 2A–C). However, the expression level of TUBA1B was significantly higher in patients with early COAD, suggesting that TUBA1B may have potential value for early diagnosis of COAD patients (Fig. 2D).

We further explored the relationship between TUBA1B expression and immune and molecular subtypes across various cancers using the TISIDB database. Our analysis categorized the tumors into 6 immune subtypes, including C1 (wound healing), C2 (IFN-gamma dominant), C3 (inflammatory), C4 (lymphocyte depleted), C5 (immunologically quiet), and C6 (TGF-b dominant), based on their immune activity and observed significant associations between TUBA1B expression and immune subtypes in 18 different cancers, including BLCA, BRCA, COAD, ESCA, HNSC, KIRP, LGG, LIHC, LUAD, LUSC, OV, PAAD, PCPG, PRAD, SARC, STAD, THCA, and UCEC. Across most cancers, TUBA1B expression tended to be lowest in the C3 immune subtype and highest in the C1, C2, and C4 immune subtypes (Fig. 2E). Additionally, we assessed the differential expression of TUBA1B among various molecular subtypes, and the findings revealed varying TUBA1B expression levels in 12 distinct cancer subtypes with different molecular profiles (Fig. 2F). Specifically, TUBA1B exhibited the highest expression in the basal subtype of BRCA, HM-SNV subtype of COAD, ESCC subtype of ESCA, Basal subtype of HNSC, C2c-CIMP subtype of KIRP, Classic-like subtype of LGG, Primitive subtype of LUSC, Proliferative subtype of OV, Kinase signaling subtype of PCPG, 6-FOXA1 subtype of PRAD, HM-indel subtype of STAD, and MSI subtype of UCEC. Overall, our analysis revealed that TUBA1B expression varies across different immune and molecular subtypes within the pan-cancer landscape.

Taken together, we observed a consistent upregulation of TUBA1B expression in most cancers, which is also supported by protein-level data. Furthermore, TUBA1B expression was significantly associated with both immunophenotyping and molecular subtyping across multiple cancer types, highlighting the potential significance of TUBA1B as a pan-cancer marker with relevance to both immune and molecular classifications.

### Prognostic and diagnostic value of TUBA1B in pan-carcinoma

**Figure 3:**
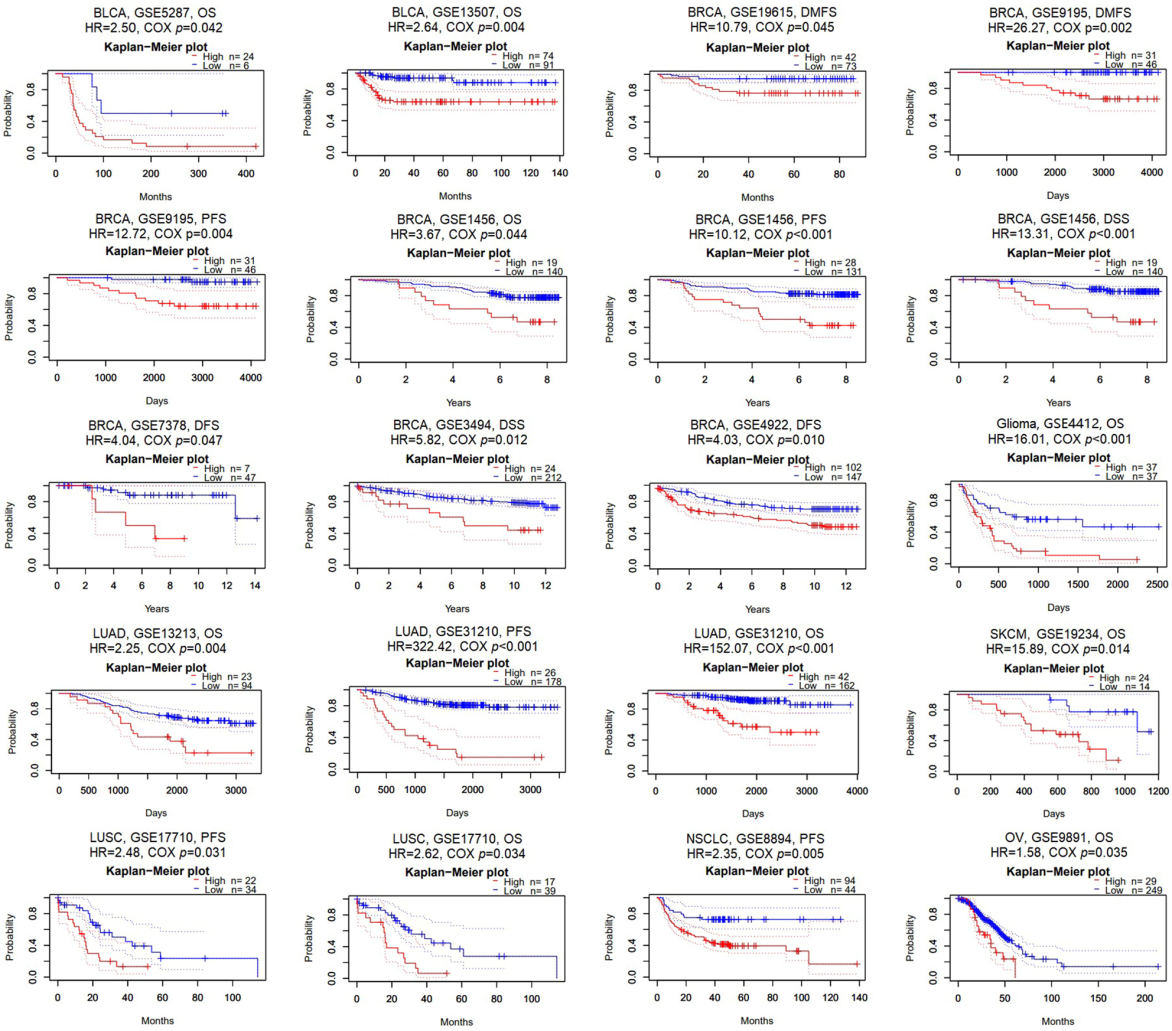
Survival analysis of TUBA1B in various GEO datasets from the PrognoScan database.

Using the PrognoScan database, we analyzed cancer patients with varying levels of TUBA1B expression to assess its impact on their prognosis in 15 datasets from diverse cancer types, including (GSE5287, GSE13507, GSE19615, GSE9195, GSE1456, GSE7378, GSE3494, GSE4922, GSE4412, GSE13213, GSE31210, GSE19234, GSE17710, GSE8894, and GSE9891) from BLCA, BRCA, glioma, LUAD, SKCM, LUSC, non-small cell lung carcinoma (NSCLC), and OV. Overall, our findings consistently demonstrated that higher TUBA1B expression correlated with poorer prognosis in these cancers, affecting various survival metrics such as overall survival (OS), progression-free survival (PFS), disease-specific survival (DSS), distant metastasis-free survival (DMFS), and disease-free survival (DFS) (Cox *p* < 0.05; Fig. 3).

**Figure 4:**
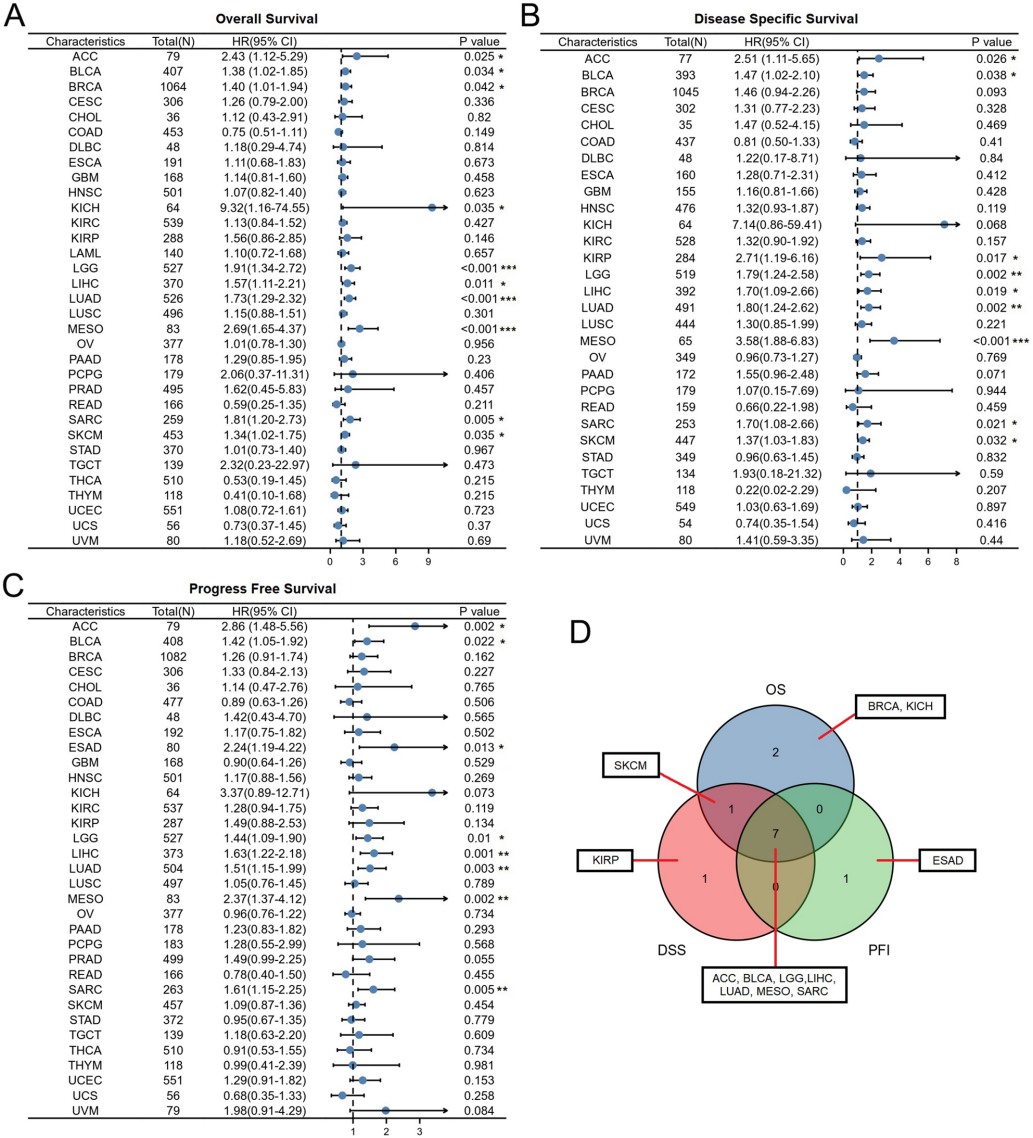
Association between TUBA1B expression and prognosis in cancer patients. (**A**) Association between TUBA1B expression and overall survival (os) in cancer patients. (**B**) Association between TUBA1B expression and disease-specific survival (DSS) in cancer patients. (**C**) Association between TUBA1B expression and progression-free survival (PFS) in cancer patients. (**D**) The Venn diagram shows the intersection of OS, DSS, and PFS for different cancers. *p < 0.05, **p < 0.01, ***p < 0.001.

Next, we used TCGA RNA-seq data to further analyze the prognostic value of TUBA1B regarding OS, DSS, and PFS. To investigate the association between TUBA1B expression and the survival outcomes of cancer patients, we categorized the cancer samples into two groups based on their TUBA1B expression levels relative to the median value. Cox regression analysis revealed that TUBA1B expression had a significant impact on OS in several cancer types, including ACC (*p* = 0.025, HR = 2.43), BLCA (*p* = 0.034, HR = 1.38), BRCA (*p* = 0.042, HR = 1.40), KICH (*p* = 0.035, HR = 9.32), LGG (*p* < 0.001, HR = 1.91), LIHC (*p* = 0.011, HR = 1.57), LUAD (*p* < 0.001, HR = 1.73), MESO (*p* < 0.001, HR = 2.69), SARC (*p* = 0.005, HR = 1.81), SKCM (*p* = 0.035, HR = 1.34). In summary, high expression of TUBA1B was identified as a risk factor for OS (Figs. 4A; S3A).

We also investigated the association between TUBA1B expression and patient DSS and discovered that high expression of TUBA1B is a risk factor for DSS in ACC (*p* = 0.026, HR = 2.51), BLCA (*p* = 0.038, HR = 1.47), KIRP (*p* = 0.017, HR = 2.71), LGG (*p* = 0.002, HR = 1.79), LIHC (*p* = 0.019, HR = 1.70), LUAD (*p* = 0.002, HR = 1.80), MESO (*p* < 0.001, HR = 3.58), SARC (*p* = 0.021, HR = 1.70), SKCM (*p* = 0.032, HR = 1.37) (Figs. 4B; S3B). Subsequently, we investigated the TUBA1B influence on patients’ PFS to investigate the possible connection between TUBA1B and the growth of tumors in patients; it was found that TUBA1B affected the PFS of the following patients: ACC (*p* = 0.002, HR = 2.86), BLCA (*p* = 0.022, HR = 1.42), ESAD (*p* = 0.013, HR = 2.24), LGG (*p* = 0.010, HR = 1.44), LIHC (*p* = 0.001, HR = 1.63), LUAD (*p* = 0.003, HR = 1.51), MESO (*p* = 0.002, HR = 2.37), and SARC (*p* = 0.005, HR = 1.61) (Figs. 4C; S3C). Collectively, we found that TUBA1B affects three prognostic indicators in ACC, BLCA, LGG, LIHC, LUAD, MESO, and SARC patients (Fig. 4D).

**Figure 5:**
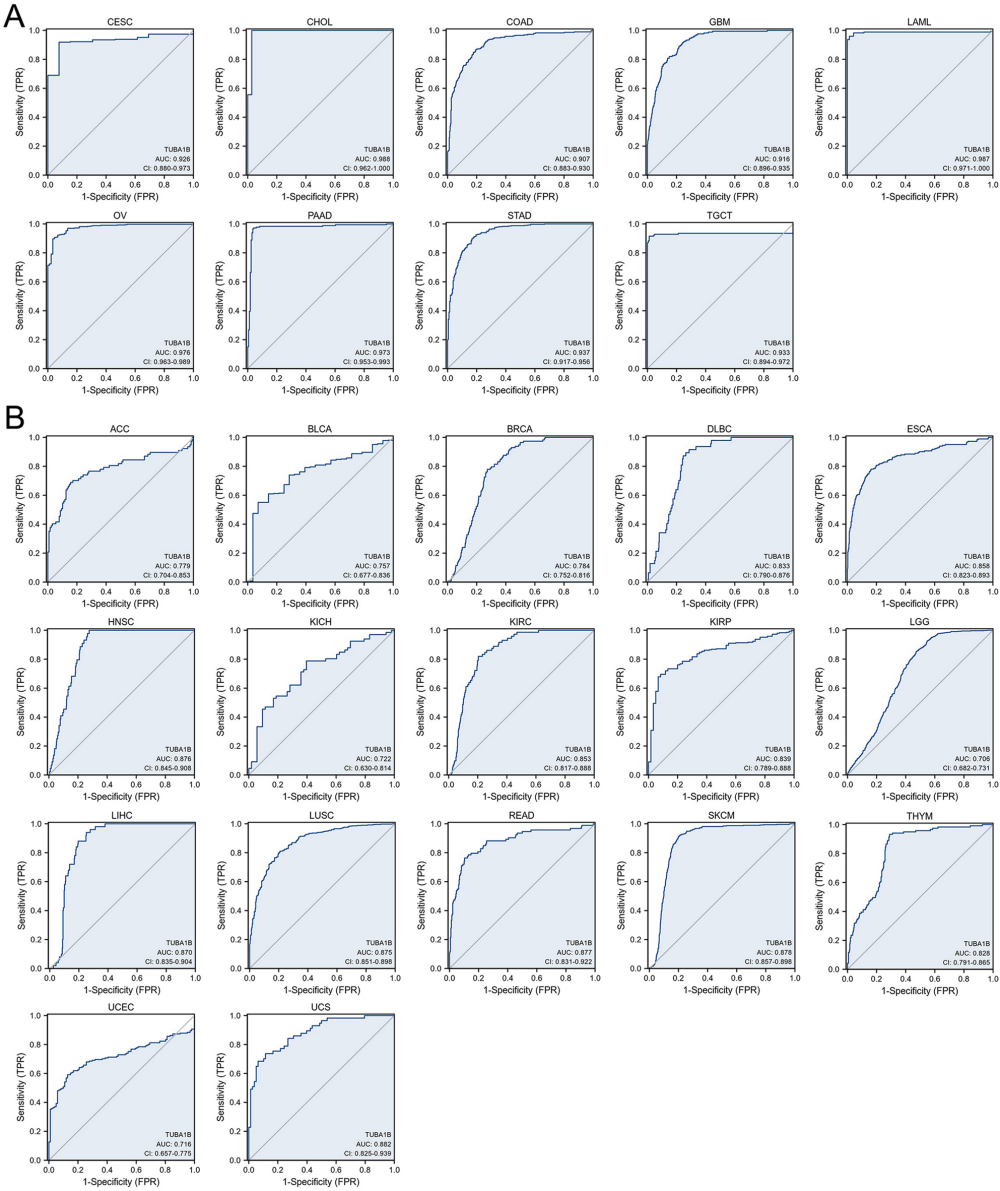
Receiver operating characteristic (ROC) curve for TUBA1B expression in pan-cancer. (**A**) TUBA1B expression in cancers with good diagnostic value (AUC > 0.9), including CESC, CHOL, COAD, GBM, LAML, OV, PAAD, STAD, and TGCT. (**B**) TUBA1B expression in cancers with some diagnostic value (AUC > 0.9), including ACC, BLCA, BRCA, DLBC, ESCA, HNSC, KICH, KIRC, KIRP, LGG, LIHC, LUSC, READ, SKCM, THYM, UCEC, and UCS.

Next, we analyzed the diagnostic potential of TUBA1B in several types of cancer. The ROC curve analysis revealed that TUBA1B had a good diagnostic ability (AUC > 0.9), including CESC (0.926), CHOL (0.988), COAD (0.907), GBM (0.916), LAML (0.987), OV (0.976), PAAD (0.973), STAD (0.937), and TGCT (0.933) (Fig. 5A). TUBA1B displayed a moderate diagnostic capacity (AUC > 0.7) in the case of several other cancer types, including ACC (0.779), BLCA (0.757), BRCA (0.784), DLBC (0.833), ESCA (0.858), HNSC (0.876), KICH (0.722), KIRC (0.853), KIRP (0.839), LGG (0.706), LIHC (0.870), LUSC (0.875), READ (0.877), SKCM (0.878), THYM (0.828), UCEC (0.716), and UCS (0.882) (Fig. 5B).

Overall, high expression of TUBA1B was associated with poor prognosis in several cancer types and had a moderate to a strong ability to predict tumors and normal tissues in most cancers.

### TUBA1B is an independent factor in the prognosis of some cancers

To understand the influence of multiple factors on the OS of cancer patients, we conducted univariate and multivariate regression analyses on ten cancer types. Cancer types with *p* < 0.1 at the time of previous univariate COX regression analysis were included in the study, including ACC, BLCA, BRCA, KICH, LGG, LIHC, LUAD, MESO, SARC, and SKCM. For ACC, multivariate analysis showed that primary therapy outcome (partial response (PR)/complete response (CR), hazard ratio (HR) = 0.108, *p* = 0.001) is the only independent prognostic factor (Table S2A). For BLCA, primary therapy outcome (PR/CR, HR = 0.382, *p* = 0.001) was the only independent prognostic factor (Table S2B). For BRCA, clinical stage (stage III/IV, HR = 3.048, *p* = 0.002), age (> 60, HR = 2.391, *p* < 0.001), radiation therapy (yes, HR = 0.427, *p* < 0.001), and TUBA1B expression (high TUBA1B, HR = 1.808, *p* = 0.009) were identified as independent prognostic factors (Table S2C). For KICH, T stage (T3/T4, HR = 11.318, *p* = 0.003) and TUBA1B expression (high TUBA1B, HR = 11.480, *p* = 0.023) were identified as independent prognostic factors (Table S2D). For LGG, the WHO grade (G3, HR = 2.696, *p* < 0.001), primary therapy outcome (PR/CR, HR = 0.243, *p* < 0.001), age (> 40, HR = 2.902, *p* < 0.001), and TUBA1B expression (high TUBA1B, HR = 1.653, *p* = 0.022) were independent prognostic factors (Table S2E). For LUAD, N stage (N1/N2/N3, HR = 1.602, *p* = 0.044), primary therapy outcome (PR/CR, HR = 0.376, *p* < 0.001), and TUBA1B expression (high TUBA1B, HR = 1.576, *p* = 0.031) were independent prognostic factors (Table S2G). For MESO, TUBA1B expression (high TUBA1B, HR = 2.689, *p* =  < 0.001) was the only independent prognostic factor (Table S2H). For SARC, residual tumor (R1, HR = 2.115, *p* = 0.017) (R2, HR = 15.315, *p* < 0.001) and metastasis (transferred group, HR = 2.377, *p* = 0.002) were independent prognostic factors (Table S2I). For SKCM, T stage (T3, HR = 2.094, *p* = 0.031) (T4, HR = 4.522, *p* < 0.001), and N stage (N2, HR = 3.071, *p* = 0.046) (N3, HR = 7.113, *p* < 0.001) were identified as independent prognostic factors (Table S2J), whereas in LIHC, no independently influencing factors were identified (Table S2F).

**Figure 6:**
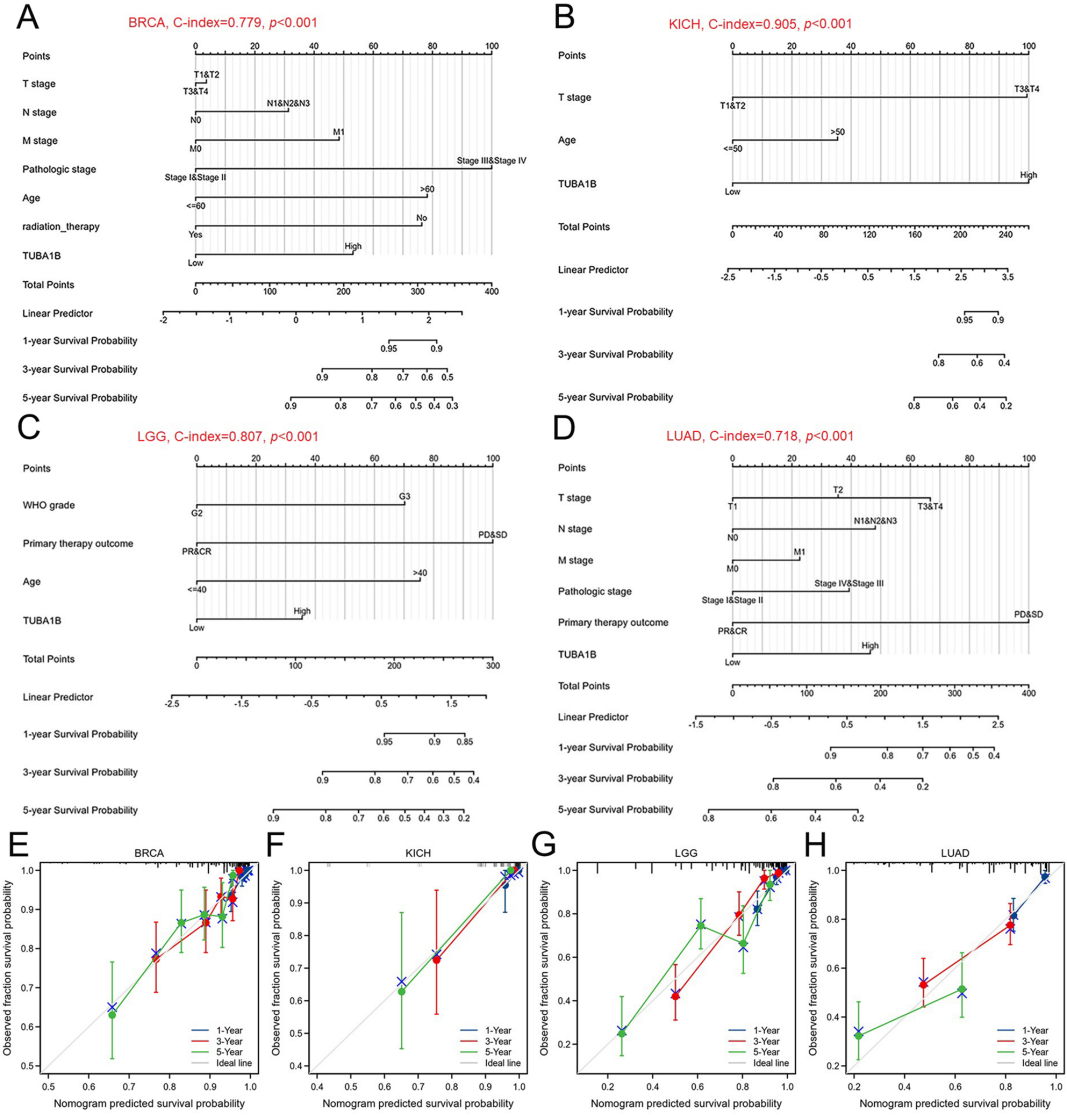
Nomograms and calibration curves predicting patient OS in four cancers. Nomograms for BRCA (**A**), KICH (**B**), LGG (**C**), and LUAD (**D**). Calibration curves for BRCA (**E**), KICH (**F**), LGG (**G**), and LUAD (**H**). The horizontal and vertical coordinates represent the model-predicted and observed survival probability, respectively. A closer alignment with the ideal line indicates better model performance.

Subsequently, we utilized variables with *p* < 0.1 from the univariate COX regression analysis to formulate prognostic nomograms and assess their calibration. Our results revealed the following concordance index (C-index) values for the nomograms: ACC (C-index = 0.870, Fig. S3A), BLCA (C-index = 0.718, Fig. S3B), BRCA (C-index = 0.779, Fig. 6A), KICH (C-index = 0.905, Fig. 6B), LGG (C-index = 0.807, Fig. 6C), LUAD (C-index = 0.718, see Fig. 6D), LIHC (C-index = 0.642, see Fig. S3C), SARC (C-index = 0.712, see Fig. S4D), and SKCM (C-index = 0.712, Fig. S3E). For MESO, where only TUBA1B emerged as an independent prognostic factor, a nomogram could not be constructed. Subsequently, calibration curves for each nomogram were generated to assess their accuracy, demonstrating close alignment with the ideal line across all the aforementioned cancer types (Figs. 6E–H and S3F–J). These findings indicate that TUBA1B can serve as a reliable independent predictor of patient outcomes in these specific tumor types.

### Analysis of genetic variation in TUBA1B

**Figure 7:**
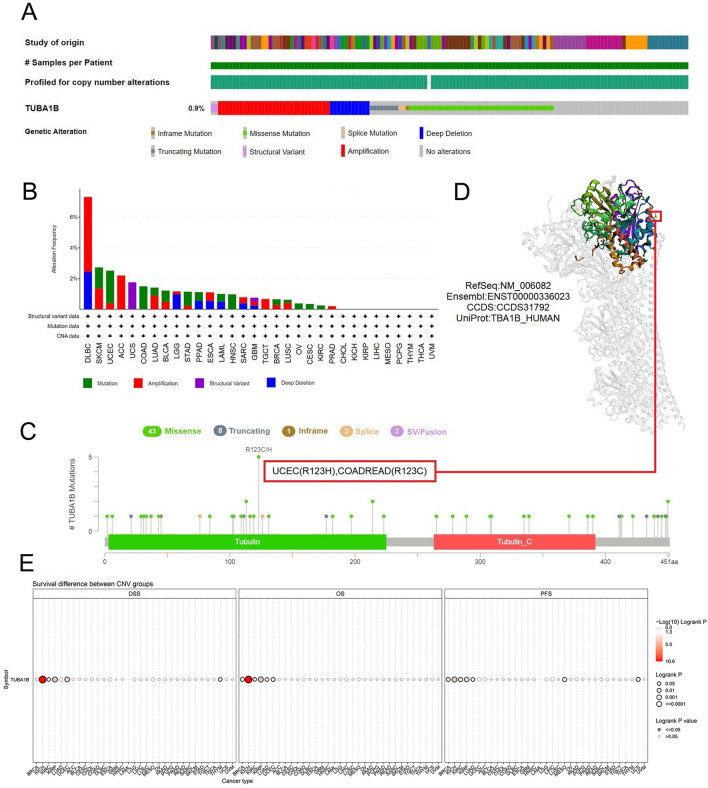
Mutated features of TUBA1B in various tumors. (**A**) Overview of TUBA1B expression alterations in different tumors. (**B**) Frequency distribution of mutation types. (**C**) Mutation sites in the TUBA1B amino acid sequences. (**D**) Visualization of select TUBA1B mutations on the 3D protein structure. (**E**) Association between CNV in TUBA1B and cancer patient prognosis.

Cancer is driven by numerous types of genetic changes, some of which could be potential molecular therapeutic targets^28^. To investigate whether or not TUBA1B can be used as a molecular therapeutic target, we explored its genetic alterations. The results showed that of the 10,443 samples, 95 (0.9%) had the TUBA1B mutation. Among them, the most important CNV mutations are missense mutations, followed by amplification, deep deletion, and truncating mutations (Fig. 7A), with 44.35% of the mutations categorized as missense substitutions, while 26.21% were synonymous substitutions (Fig. S4A). Additionally, the most prevalent SNV categories included G > A (25.99%), C > T (25.42%) and A > G (12.99%) (Fig. S4B). Among the cancer types, DLBC (7.32%), SKCM (2.73%), UCEC (2.51%), ACC (2.2%), and UCS (1.75%) exhibited the highest mutation frequencies (Fig. 7B). Notably, the Tubulin/FtsZ domain had the most frequent mutation sites, predominantly featuring R123C/H mutations, with two occurrences in UCEC (R123H) and three in COAD/READ (R123C) (Fig. 7C). Lastly, we provide a comprehensive visualization of the mutations within the 3D structure and sequence of the TUBA1B protein (Fig. 7D).

The GSCA database serves as a pivotal resource in cancer research as it encompasses a wide array of gene set analyses, ranging from genomics, and pharmacogenomics to immunogenomics. Notably, CNV pie charts derived from this database reveal prevalent patterns, indicating that most cancers exhibit heterozygous amplifications and deletions, with occasional rare occurrences of homozygous amplifications in DLBC, CHOL, ACC, SKCM, and sporadic homozygous deletions in DLBC and LGG (Fig. S4C). Moreover, our investigation showed a significant positive correlation between TUBA1B expression and CNV alterations across diverse malignancies (Fig. S4D). Additionally, CNV in TUBA1B emerged as a prognostic risk factor affecting patients’ outcomes in BRCA, KICH, KIRC, KIRP, LUAD, and UCEC (Fig. 7E).

### Correlation analysis of TUBA1B with methylation

**Figure 8:**
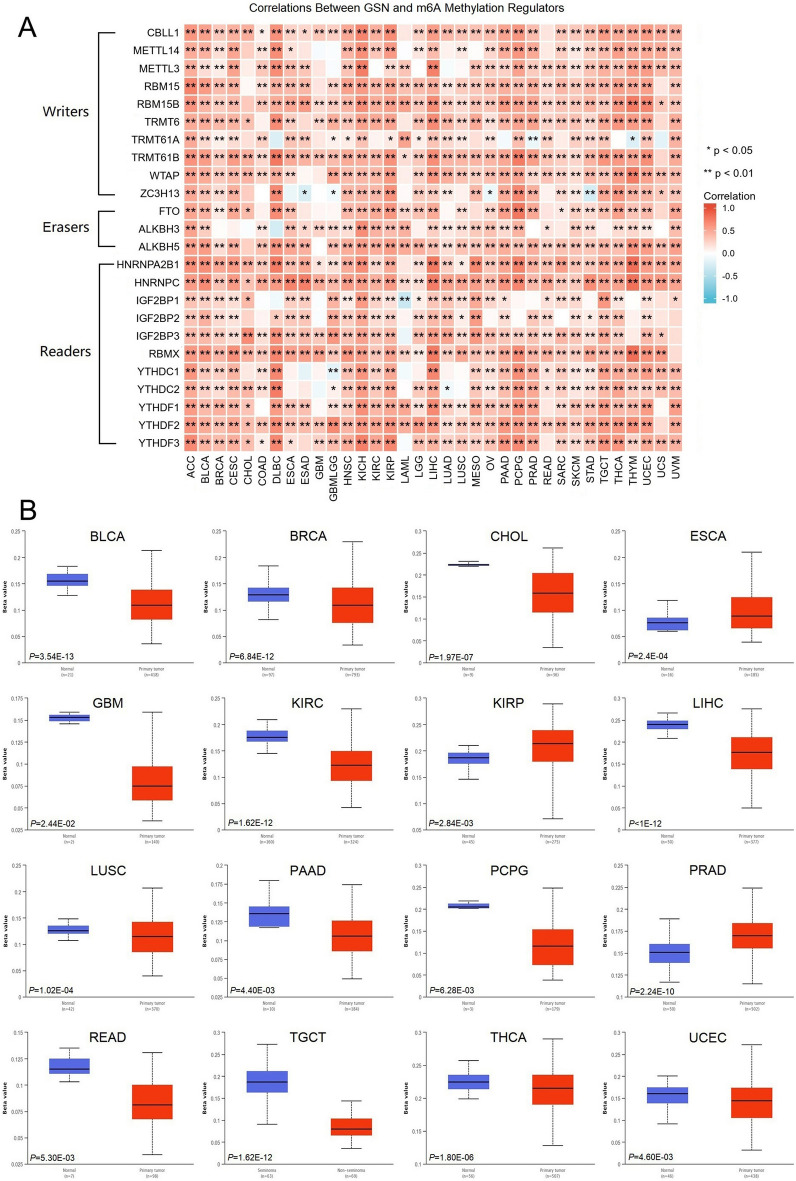
Epigenetic methylation analysis of TUBA1B. (**A**) Relationship between TUBA1B mRNA expression and m6A methylation regulators in various cancers. (**B**) Differential promoter methylation levels of TUBA1B in normal tissues and tumors based on UALCAN data (β values).

There is a growing body of evidence highlighting the significance of abnormal methylation in cancer development, offering potential insights for innovative cancer treatments^29^. Herein, we assessed the relationship between TUBA1B expression and key m6A methylation regulators, encompassing 24 essential regulators, including 10 writers (CBLL1, METTL14, METTL3, RBM15, RBM15B, TRMT6, TRMT61A, TRMT61B, WTAP, and ZC3H13), 3 erasers (FTO, ALKBH3, and ALKBH5), and 11 readers (HNRNPA2B1, HNRNPC, IGF2BP1, IGF2BP2, IGF2BP3, RBMX, YTHDC1, YTHDC2, YTHDF1, YTHDF2, and YTHDF3). Our heatmap analysis reveals that, in most cancer types, TUBA1B expression positively correlates with the expression of these m6A methylation-related genes (Fig. 8A). Given the widespread overexpression of TUBA1B in various cancers, we propose that it may also exhibit heightened levels of m6A methylation.

Using the UALCAN database, we conducted a comparative analysis of TUBA1B promoter methylation levels between normal and tumor tissues. Our findings revealed that TUBA1B promoters exhibited hypo-methylation in various cancers (β < 0.25) (Fig. 8B). Among them, compared with normal tissues, TUBA1B promoters’ methylation levels were found to be lower in BLCA (*p* = 3.54E-13), BRCA (*p* = 6.84E-12), CHOL (*p* = 1.97E-07), GBM (*p* = 2.44E-02), KIRC (*p* = 1.62E-12), LIHC (*p* < 1E-12), LUSC (*p* = 1.02E-04), PAAD (*p* = 4.40E-03), PCPG (*p* = 6.28E-03), READ (*p* = 5.30E-03), TGCT (*p* = 1.62E-12), THCA (*p* = 1.80E-06), UCEC (*p* = 4.60E-03) cancerous tissues (Fig. 8B). Conversely, the methylation level of the TUBA1B promoter in ESCA (*p* = 2.4E-04), KIRP (*p* = 2.84E-03), and PRAD (*p* = 2.24E-10) in cancer tissues were higher than in their corresponding normal tissues (Fig. 8B). Subsequently, we analyzed the effect of TUBA1B methylation levels on the prognosis of cancer patients, including DSS, OS, and PFS, and the results showed that the low methylation status of TUBA1B was a risk factor for patients with LGG and LIHC, but a protective factor for patients with UCEC (Fig. S4E).

In summary, our findings indicate that TUBA1B is highly expressed in most cancers and positively correlated with the expression of most m6A methylation-related genes. Thus, we hypothesize that TUBA1B exhibits high methylation levels in most cancers and TUBA1B promoters are hypomethylated. TUBA1B methylation levels also affect the prognosis of cancer patients.

### Relationship between TUBA1B expression and immunity

**Figure 9:**
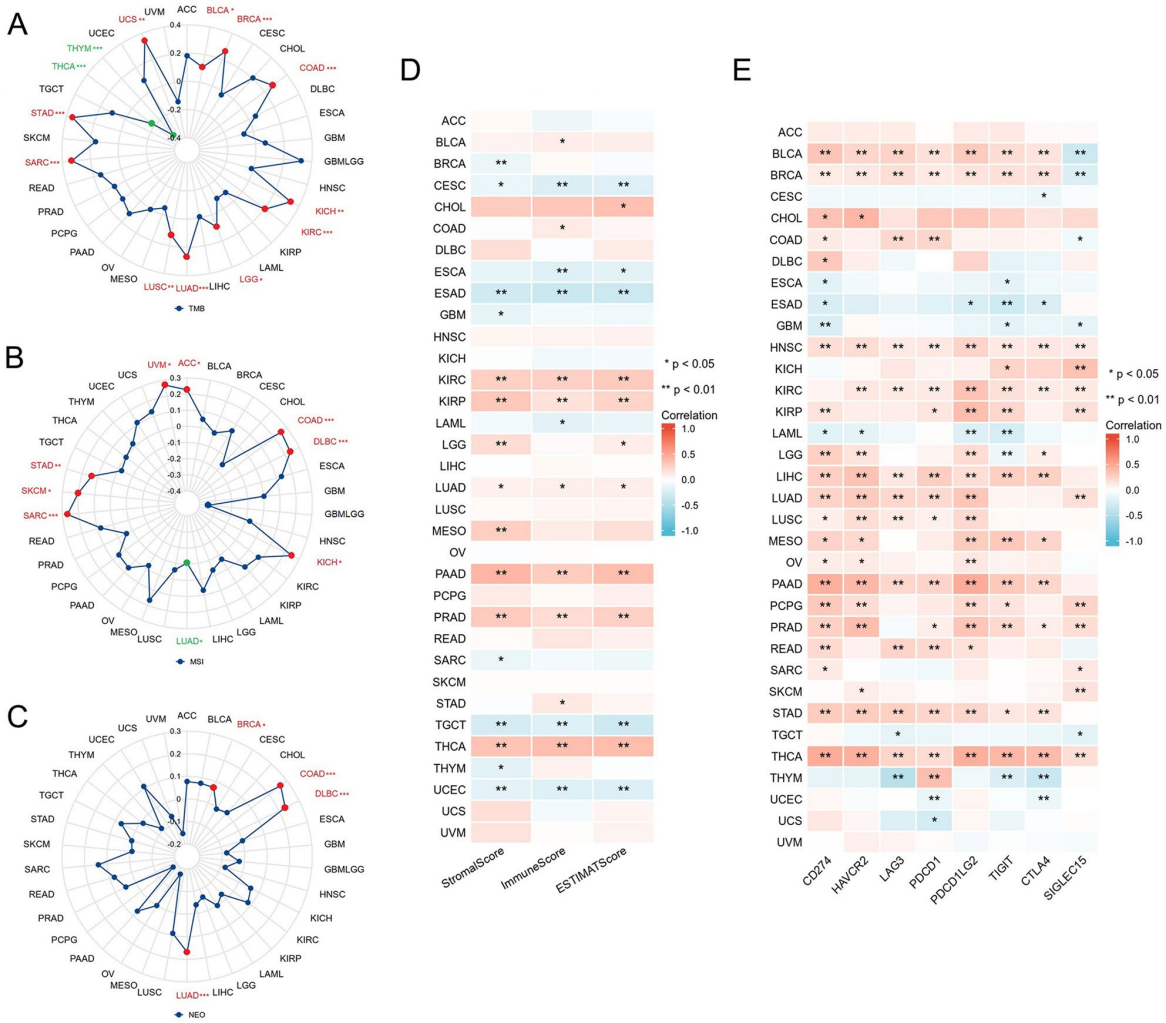
Association of TUBA1B expression with TMB, MSI, NEO, TME, and immune checkpoints in 33 cancer types. (**A**) Correlation between TUBA1B expression and TMB, MSI, and NEO in 33 cancers. (**B**) Correlation between TUBA1B expression and StromalScore, ImmuneScore, and ESTIMATEScore in 33 cancers. (**C**) Correlation between TUBA1B expression and immune checkpoint expression in 33 cancers. (*p < 0.05, **p < 0.01, ***p < 0.001).

Considering that TMB, MSI, and NEO are all considered potential predictive biomarkers for immunotherapy response^30^, we explored the relationship between TUBA1B expression levels and these biomarkers and identified significant correlations between TUBA1B expression and TMB in 14 cancer types. Specifically, TUBA1B expression exhibited a positive correlation with TMB in BLCA, BRCA, COAD, GBMLGG, KICH, KIRC, LGG, LUAD, LUSC, SARC, STAD, and UCS. Conversely, in THCA and THYM, a negative correlation was observed between TUBA1B expression and TMB (Fig. 9A). In 10 cancer types, we observed significant correlations between TUBA1B expression and MSI. Notably, TUBA1B expression positively correlated with MSI in ACC, COAD, DLBC, KICH, SARC, SKCM, STAD, and UVM, while in GBMLGG, a negative correlation was found (Fig. 9B). Additionally, we identified a positive association between TUBA1B expression and NEO solely in BRCA, COAD, DLBC, and LUAD (Fig. 9C).

Next, we evaluated the relationship between TUBA1B expression and matrix and immune scores across various cancers using the ESTIMATE algorithm, and the results revealed that TUBA1B expression was positively correlated with Stromal Score, Immune Score, and ESTIMATE Score in GBMLGG, KIRC, KIRP, LUAD, PAAD, PRAD, and THCA. Conversely, in CESC, ESAD, TGCT, UCEC, and LUADLUSC, TUBA1B expression exhibited inverse correlations with Stromal Score, Immune Score, and ESTIMATE Score (Fig. 9D). Furthermore, we explored the association between TUBA1B expression and immune checkpoint expression, including CD274, HAVCR2, LAG3, PDCD1, PDCD1LG2, CTLA4, SIGLEC15, and TIGIT. Our heatmap analysis demonstrated that TUBA1B expression was significantly positively correlated with most immune checkpoints in BLCA, BRCA, HNSC, KIRC, KIRP, LIHC, LUAD, LUSC, MESO, PAAD, PRAD, STAD, and THCA (Fig. 9E). Additionally, we examined the relationship between TUBA1B expression and immune-related genes obtained from the GSEA database, which included 43 immune activation-related genes and 22 immunosuppression-related genes. Across most cancer types, TUBA1B expression exhibited positive correlations with most immune-related genes. Furthermore, the expression of CD276, MICB, PVR, TGFB1, and TGFBR1 displayed high correlations with TUBA1B expression in most cancers (Fig. S5).

**Figure 10:**
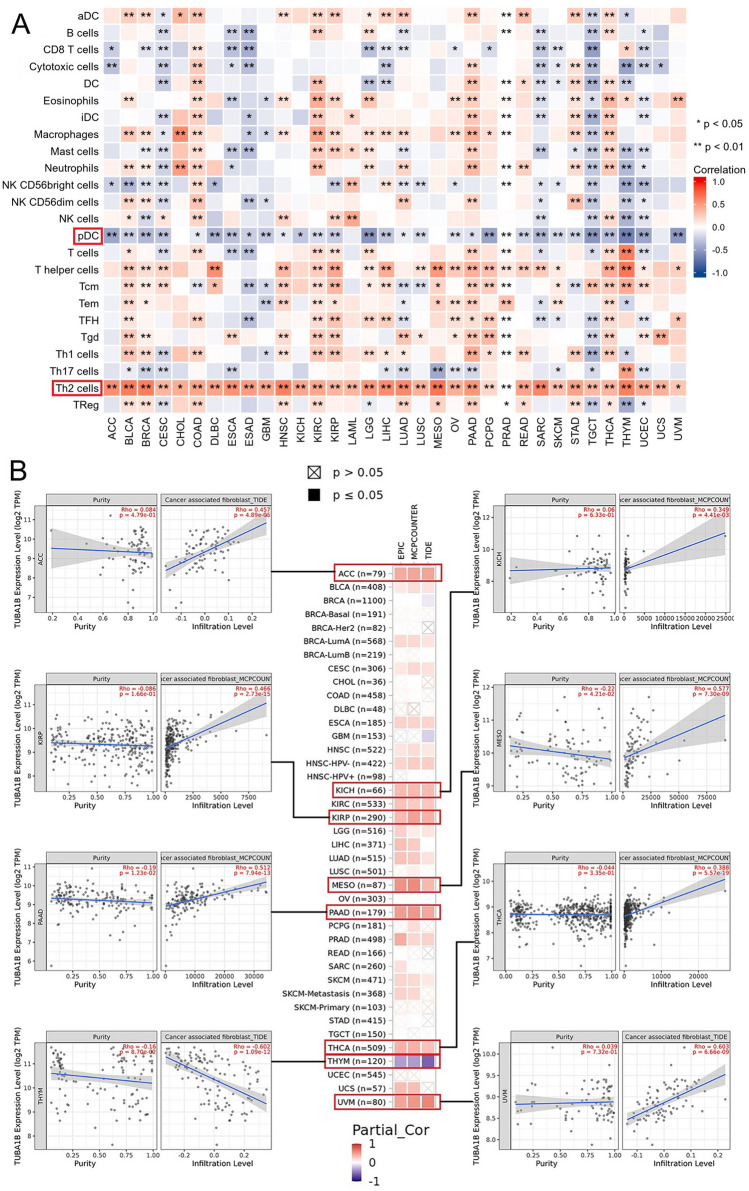
Relationships between immune cell infiltration levels and TUBA1B expression in pan-cancer. (**A**) Correlation between TUBA1B expression and immune infiltration using the ssGSEA algorithm. (**B**) Correlation analysis of TUBA1B expression with immune infiltration of CAF cells based on the Timer2.0 database. Scatter plots include ACC, KICH, KIRP, MESO, PAAD, THCA, THYM, and UVM. (*p < 0.05, **p < 0.01).

TIICs are an important part of TME and are closely related to the occurrence, development, and metastasis of cancer. Therefore, we used the ssGSEA algorithm to evaluate the correlation between TUBA1B expression and the degree of infiltration of 24 immune cells. The heat map results showed that in most cancers, TUBA1B expression was negatively correlated with pDC but significantly positively correlated with tumor-promoting Th2 cells (Fig. 10A). Next, we analyzed the correlation between TUBA1B expression levels and immune cell infiltration in various tumors using the EPIC, MCP-COUNTER, CIBERSORT, and TIDE algorithms in the Timer2.0 database. The results showed that the expression of TUBA1B was positively correlated with the degree of infiltration of CAFs in ACC (*r* = 0.457, *p* = 4.89E-05), KICH (*r* = 0.349, *p* = 4.41E-03), KIRP (*r* = 0.466, *p* = 42.73E-15), MESO (*r* = 0.577, *p* = 7.30E-09), PAAD (*r* = 0.512, *p* = 7.94E-13), THCA (*r* = 0.388, *p* = 5.57E-19) and UVM (*r* = 5.57E-19, *p* = 6.66E-09) (Fig. 10B) but negatively correlated with the degree of infiltration of CAFs in THYM (*r* = -0.602, *p* = 1.09E-12) (Fig. 10B). In addition, we found that in THYM, the expression of TUBA1B had a significant correlation with the degree of infiltration of most immune cells, including positive correlation with CD8 + T cells, CD4 + T cells, Tregs and negative correlation with macrophages and NKs (Fig. S8). For most cancers, TUBA1B expression was significantly positively correlated with the degree of invasion of myeloid-derived suppressor cells (MDSCs) (Fig. S8).

### Functional enrichment analysis of TUBA1B

**Figure 11:**
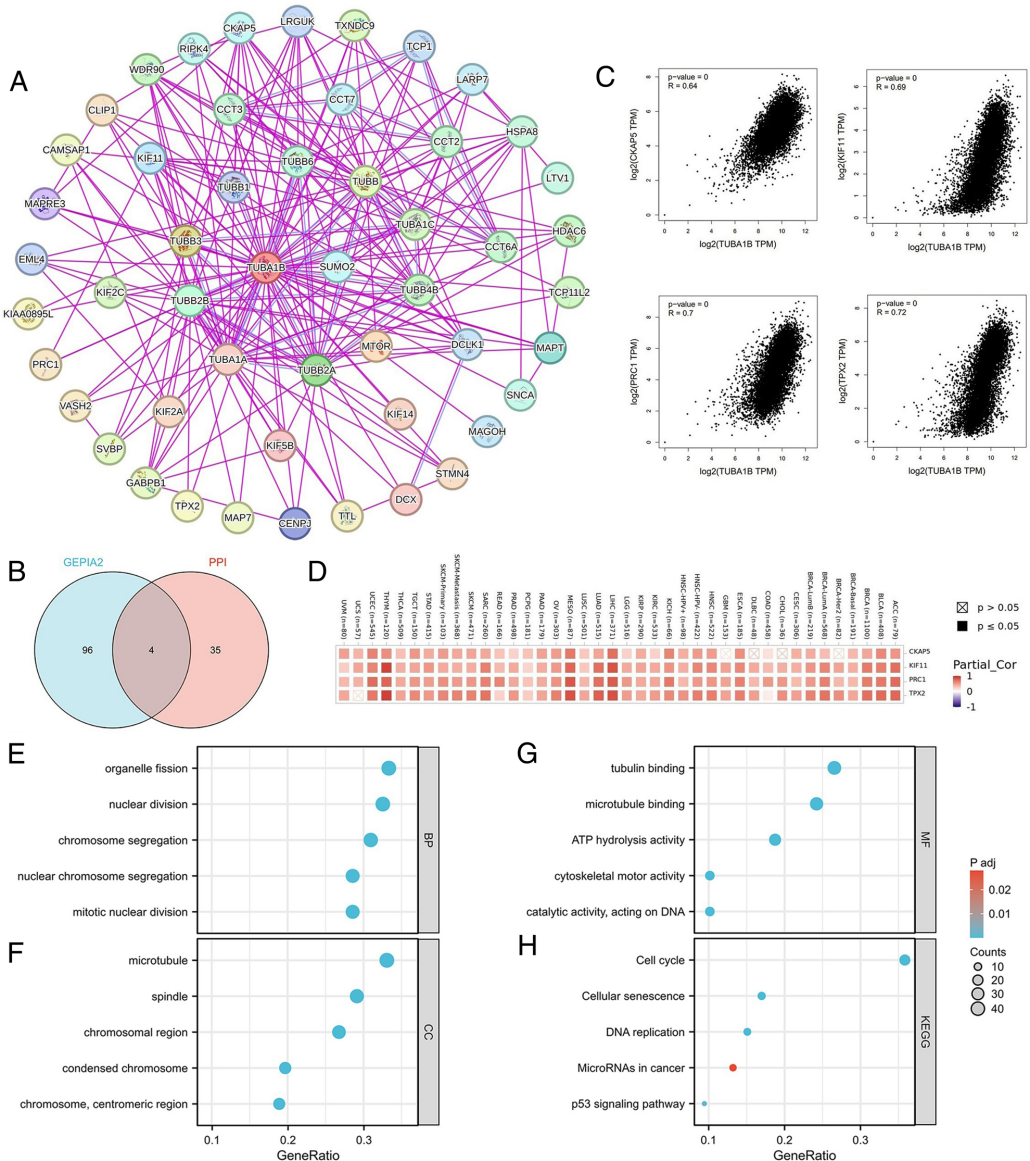
TUBA1B-related genes, interacting proteins, and functional enrichment analysis. (**A**) Protein–protein interaction (PPI) network for TUBA1B. (**B**) Venn diagram showing the intersection of TUBA1B-binding and interacting genes after selection. (**C**) and (**D**) depict the correlation between TUBA1B and six associated genes in 33 cancers. GO analysis includes biological processes (**E**), cellular components (**F**), molecular functions (**G**), and KEGG pathways (**H**).

To gain deeper insights into the biological role of TUBA1B across various cancers, we conducted further enrichment analyses. First, we compiled a list of 40 TUBA1B-binding proteins from the STRING database and constructed a Protein–Protein Interaction (PPI) network (Fig. 11A). Subsequently, we identified the top 100 genes correlated with TUBA1B from the GEPIA2 database (Table S3). Among these, CKAP5, KIF11, PRC1, STMN1, TPX2, and TUBB emerged as common members between the binding proteins and the correlated genes (Fig. 11B). Scatterplot analysis revealed a positive correlation between TUBA1B expression and the expression of CKAP5 (*r* = 0.64), KIF11 (*r* = 0.69), PRC1 (*r* = 0.7), STMN1 (*r* = 0.7), TPX2 (*r* = 0.72), and TUBB(*r* = 0.72) (Fig. 11C). In addition, the expression of TUBA1B was positively correlated with the expression of the above 6 genes in most cancers (Fig. 11D).

**Figure 12:**
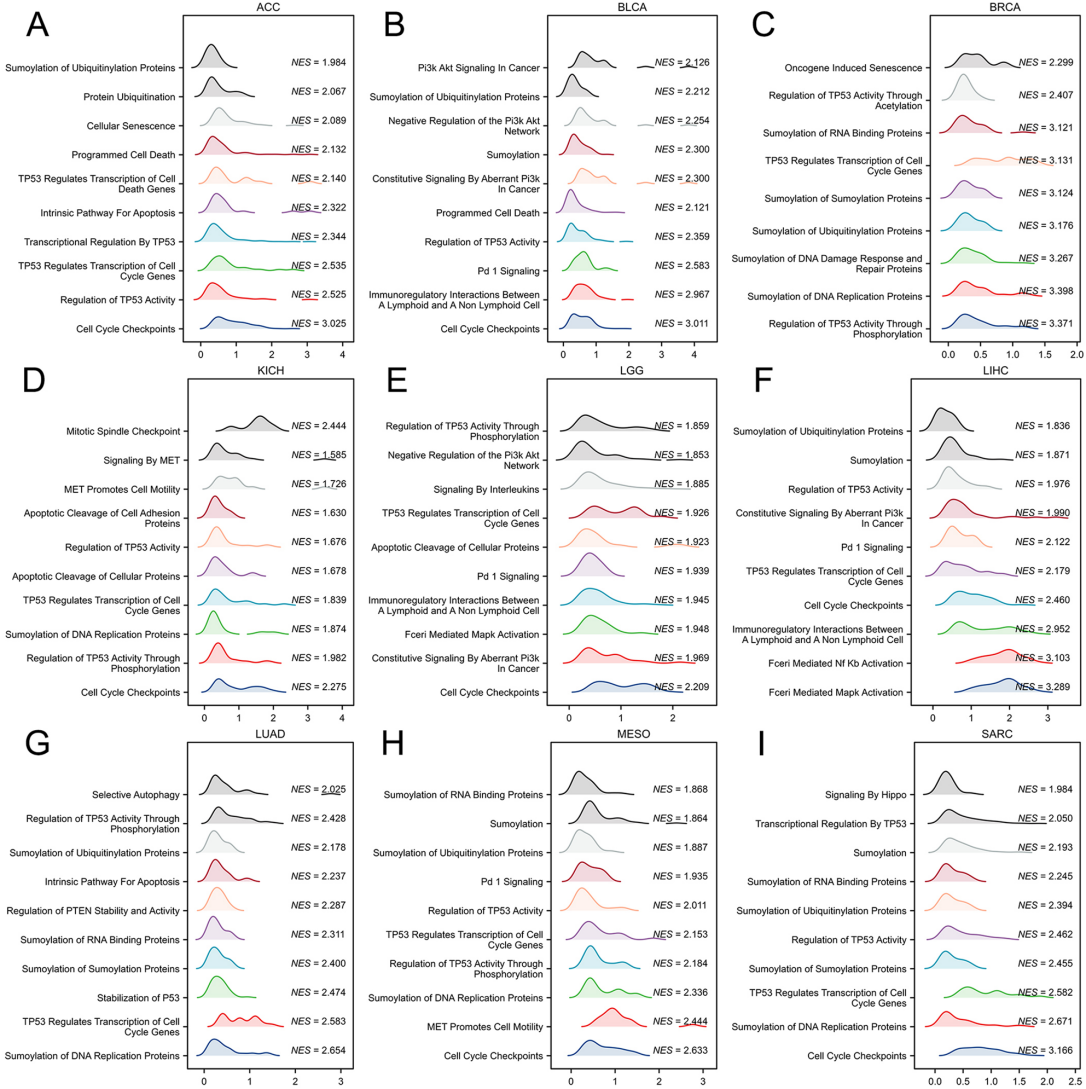
GSEA Functional Enrichment Analysis of TUBA1B in Eight Cancers. In ACC (**A**), BLCA (**B**), BRCA (**C**), LGG (**D**), LGG (**E**), LIHC (**F**), LUAD (**G**), MESO (**H**), and SARC (**I**), the first 10 pathways are positively correlated with TUBA1B expression.

Subsequently, we conducted comprehensive GO/KEGG enrichment analyses using TUBA1B binding and related genes to elucidate the biological functions associated with TUBA1B, which revealed a total of 357 GO categories, comprising 357 biological processes (BP), 64 cellular components (CC), 61 molecular functions (MF) and 22 KEGG pathways (Table S4). Notably, in cancer-related GO terms, TUBA1B was implicated in regulating the cell cycle transition phase, cell cycle checkpoint, signal transduction mediated by p53 class mediator, histone modification, and aging, as identified in the GO-BP analysis (Fig. 11E). In the GO-CC analysis, TUBA1B-related genes were enriched in microtubules, chromosomal regions, spindle structures, and centromeric regions (Fig. 11F). Furthermore, in the GO-MF analysis, TUBA1B-related genes exhibited potential involvement in tubulin binding, ATPase activity, GTP binding, and ubiquitin-like protein ligase binding functions (Fig. 11G). Our KEGG pathway analysis indicated that TUBA1B might play a role in regulating the cell cycle, gap junctions, DNA replication, cellular senescence, and the p53 signaling pathway (Fig. 11H). Utilizing the Reactome pathway database, we conducted GSEA analyses to explore potential pathways associated with TUBA1B involvement across nine cancer types, including ACC, BLCA, BRCA, KICH, LGG, LIHC, LUAD, MESO, and SARC. In Fig. 12A–I, our GSEA results revealed that genes positively correlated with TUBA1B expression were predominantly enriched in pathways related to cell cycle regulation, cell senescence, programmed cell death, TP53 activity regulation, immune-related pathways, PD-1 signaling, negative regulation of the PI3K/AKT pathway, and the sumoylation modification of various proteins.

Based on these findings, it could be deduced that TUBA1B plays an important role in cancer, mainly by influencing cell cycle, cell senescence, programmed death, TP53 pathway regulation, immune-related pathways, etc.

### Validation of the expression level of TUBA1B in breast cancer tissues and the phenotypic regulation function of cancer cells

**Figure 13:**
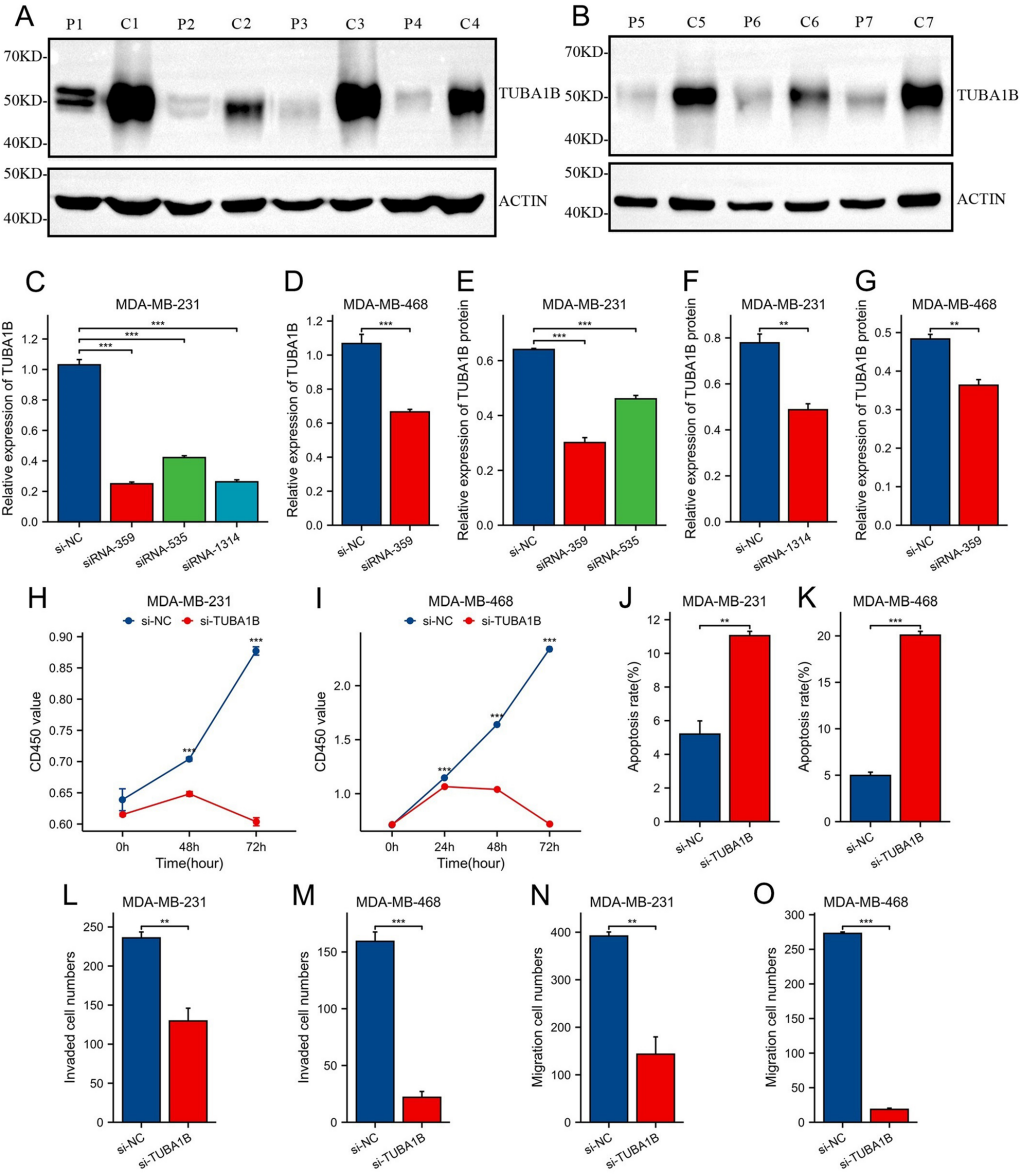
Expression levels of TUBA1B in paired breast cancer tissues, knockdown of TUBA1B inhibiting proliferation, migration, and invasion of breast cancer cells, and promoting apoptosis. (**A**, **B**) TUBA1B protein expression in breast cancer and adjacent tissues. Original blots are presented in Fig. S9. (**C**) Analysis of knockdown efficiency of TUBA1B transfected with siRNA-359, siRNA-535, and siRNA-1314 in MDA-MB-231 cell lines. (**D**) Knockdown efficiency of TUBA1B transfected with siRNA-359 in MDA-MB-468 cell line. (**E**) Detection of TUBA1B protein expression in MDA-MB-231 cell line transfected with siRNA-359 and siRNA-535. (**F**) Expression of TUBA1B protein in MDA-MB-231 cell line transfected with siRNA-1314. (**G**) Expression of TUBA1B protein in MDA-MB-468 cell line transfected with siRNA-359. (**H**, **I**) Knockdown of TUBA1B inhibited the proliferation of breast cancer cell lines. (**J**, **K**) Knockdown of TUBA1B promoted apoptosis of breast cancer cell lines. (**L**, **M**) Knockdown of TUBA1B inhibited the invasion of breast cancer cell lines. (**N**, **O**) Knockdown of TUBA1B inhibited the metastasis of breast cancer cell lines. (**p < 0.01, ***p < 0.001).

To explore the functional role of TUBA1B in breast cancer cells, we initially assessed its expression in adjacent normal tissues and breast cancer tissues. Our results revealed a significant upregulation of TUBA1B expression in breast cancer tissues compared to adjacent normal tissues (Fig. 13A, B). Subsequently, we developed three siRNA-based TUBA1B knockdown vectors and transfected them into two selected human breast cancer cell lines, MDA-MB-231 and MDA-MB468. The efficiency of TUBA1B knockdown was confirmed through RT-PCR and Western blot analyses. In addition, we observed that all three siRNA TUBA1B knockdown vectors effectively suppressed TUBA1B expression, with siRNA-359 demonstrating the highest knockdown efficiency (Figs. 13C–G; S7A). As a result, siRNA-359 was chosen as the plasmid vector for further investigations.

To investigate the association between TUBA1B and breast cancer cell proliferation, we conducted CCK-8 assays to assess cell proliferation and viability in breast cancer cells. Transfection of si-TUBA1B into the selected breast cancer cell lines at various time points (24 h, 48 h, 72 h) resulted in a significant reduction in cell proliferation and viability (Figs. 13H–I; S7B–C). Furthermore, TUBA1B knockdown was found to enhance apoptosis in breast cancer cells (Figs. 13J–K, S7D–E), providing additional evidence that TUBA1B knockdown effectively regulated the number of breast cancer cells. Subsequent Transwell and invasion assays were used to validate the impact of TUBA1B on the migratory and invasive capabilities of the selected breast cancer cells. The results indicated a significant reduction in the invasion and migration abilities of breast cancer cells following TUBA1B knockdown (Figs. 13L–O; S8A–D). Taken together, our study demonstrated that knockdown of TUBA1B inhibited the proliferation, invasion, and migration, and increased apoptosis of breast cancer cells.

## Discussion

TUBA1B is an important component subtype of α-tubulin. To our knowledge, this is the first pan-cancer investigation of TUBA1B to shed light on the expression, function, and gene mechanism across various malignancies to identify novel therapeutic approaches and targets for enhancing cancer treatment outcomes. TUBA1B is situated on human chromosome 12q13.12, and genetic alterations on chromosome 12q have been observed in a broad spectrum of malignancies, including NSCLC, leukemia, and others^31,32^. Furthermore, studies have revealed the proximity of TUBA1B to the signal transducer and activator of the transcription 6 (STAT6) locus, which has been associated with various solid tumors^33^. Our study highlights that TUBA1B is significantly overexpressed in most cancers compared to normal samples and strongly linked to poor prognosis, particularly in cancers such as ACC, BLCA, BRCA, KICH, LGG, LIHC, LUAD, MESO, and SARC. Interestingly, we observed that the difference in TUBA1B expression was more pronounced in advanced stages of ACC, LIHC, and LUAD, suggesting its potential relevance to the prognosis of patients with these tumors. While Hu et al.^13^ reported a negative impact of low TUBA1B expression on OS in patients with COAD, such was not observed in this study, possibly because this study did not encompass all COAD samples from the TCGA database. Nevertheless, our findings did reveal a decline in TUBA1B expression with increasing stages of COAD patients, suggesting that TUBA1B may serve as a promising early diagnostic marker for COAD, even though its prognostic value requires further investigation in future studies.

This study further validated the expression of TUBA1B in paired BRCA samples using clinical surgical specimens, which aligned with the above results. BRCA tissues exhibited a significantly higher expression of TUBA1B protein compared to adjacent tissues. Additionally, previous articles have reported a combination of NRF1, TUBA1B, BAX, EFNA4, and BTRC as predictors with 100% certainty for the diagnosis of TNBC in black individuals^34^. Similarly, our findings indicate that TUBA1B may serve as a diagnostic marker across various cancers. Furthermore, we observed associations between TUBA1B expression and molecular subtypes in multiple cancers. Interestingly, TUBA1B expression tended to be lowest in the C3 immune subtype in most cancers, consistent with previous studies highlighting the favorable survival outcomes associated with the C3 immune subtype^35^. Additionally, we identified TUBA1B as an independent prognostic factor for patients with BRCA, KICH, LGG, LUAD, and MESO, marking a significant step toward its potential application in future cancer management.

Epigenomic dysregulation is known to induce aberrant transcriptional processes, impacting tumor immunogenicity and the antitumor immune response, ultimately contributing to cancer development and progression^36,37^. In our study, we observed the presence of TUBA1B mutations in most tumors, which were associated with increased TUBA1B expression. Specifically, amplification mutations of TUBA1B were linked to elevated TUBA1B expression in the cancer tissues of patients with BRCA, KIRC, KIRP, and LUAD and may serve as a risk factor affecting the prognosis of patients with these types of cancer. Thus, TUBA1B mutations may play a significant role in the initiation and progression of cancer, and our study is the first to shed light on the importance of TUBA1B mutations in a pan-cancer context. However, further research is needed to delve into the underlying mechanisms.

Substantial evidence suggests that TMB and MSI can predict ICI antitumor efficacy^38^, as higher TMB and MSI levels are generally associated with improved responses to ICI therapy and better overall prognosis^39,40^. Next, we investigated the link between TUBA1B expression with TMB, MSI, and NEO, and the results showed that the TUBA1B expression was positively correlated with the three scores of BRCA, KICH, LUAD, SARC, and STAD, and TUBA1B was highly expressed in the above cancers, so patients with higher TUBA1B expression had higher three scores and would have a better response to immunotherapy. Moreover, in KIRC, KIRP, LGG, LUAD, PAAD, PRAD, and THCA, TUBA1B expression was positively linked to immunological scores, most immune checkpoints, and expression of immune-related genes. Since TUBA1B is highly expressed in these tumors, it is conceivable that TUBA1B might contribute to elevated tumor immune scores and the activation of immune-related genes. Based on these findings, TUBA1B appears to play a role in regulating cancer immunity, and targeting TUBA1B could potentially become a novel strategy for tumor immunotherapy.

The TME is composed of various immune cell infiltrations, including CAF, CD8 + T cells, CD4 + T cells, Tregs, B cells, macrophages, and NK cells. Among these, CAFs are known to play a significant role in the TME, actively contributing to cancer progression through complex interactions with various other cell types within the tumor microenvironment^41^. Similarly, MDSCs have been shown to protect cancer cells from the immune system and immunotherapy, thereby promoting cancer growth^42^. In this present study, we observed a positive correlation between TUBA1B expression and CAFs and MDSCs, both of which are implicated in immune evasion or suppression within the tumor microenvironment. Additionally, we found that TUBA1B exhibited a stronger correlation with tumor-promoting Th2 cells than with antitumor Th1 cells, suggesting a potential skewing of immune responses from antitumor to tumor-promoting in the presence of high TUBA1B expression^43^.

m6A methylation is intimately associated with cancer cell proliferation, metastasis, immune response, and other processes and affects the sensitivity and resistance of anticancer therapeutic drugs^44^. In this study, we explored the intricate relationship between TUBA1B expression and various aspects of cancer biology, including m6A methylation-related gene expression and TUBA1B promoter methylation levels. Our findings revealed a positive correlation between TUBA1B expression and the expression of m6A methylation-related genes in numerous malignancies, with ACC, BLCA, KICH, KIRP, LIHC, SKCM, and UCEC showing particularly strong associations, thereby suggesting that TUBA1B may exhibit elevated levels of m6A methylation in these cancer types. DNA methylation, which includes the addition of methyl groups to DNA molecules to regulate gene expression, plays a pivotal role in cancer drug resistance, immune responses, and other critical mechanisms^45–47^. Interestingly, our study aligns with previous research by demonstrating that reduced promoter methylation of TUBA1B is linked to the development and progression of LIHC^48^. Furthermore, our analysis revealed that increased methylation of TUBA1B is correlated with improved prognosis in patients with LGG, LIHC, and KIRP, thereby highlighting the significance of increased TUBA1B methylation in cancer development and progression.

The Tubulin/FtsZ domain represents a pivotal structural component in TUBA1B, serving as the foundation upon which microtubules rely for their integrity and function^49^. Moreover, the TTC5 binding site located at the terminal region of the TUBA1B protein is implicated in regulating the co-translational degradation of tubulin mRNA and exerts influence over the mitotic phase of cells^50^. Additionally, our analysis also suggests a potential involvement of TUBA1B in the signal transduction of the p53 pathway. The role of p53 is highly significant in the initiation and progression of cancer, and targeting p53 pathway modulators holds considerable promise in contemporary cancer treatment strategies^51^. In addition, existing studies have shown that the chemotherapy drug taxane can inhibit cell division by stabilizing β-tubulin by disrupting the mitotic spindle^52^. However, the role of TUBA1B in cancer is still unclear, so we further confirmed using cell experiments that knockdown α-tubulin TUBA1B can inhibit the proliferation, invasion, and migration of breast cancer cells, and increase apoptosis. This provides a new idea for an in-depth understanding of the role of α-tubulin and the development of new cancer treatments for it.

## Conclusion

In conclusion, this study represents the first comprehensive pan-cancer investigation of the TUBA1B oncogene, encompassing various omics analyses, prognostic assessments, epigenetic evaluations, methylation investigations, immune-related assays, and enrichment analyses. Furthermore, we validated the expression and functional relevance of TUBA1B in breast cancer. Collectively, these findings not only enhance our understanding of the role played by TUBA1B in carcinogenesis but also provide novel insights that can potentially advance cancer immunotherapy strategies.

### Supplementary Information

Supplementary Information.

Supplementary Table 3.

Supplementary Table 4.

## Data Availability

The original contributions presented in the study are included in the article/supplementary material. Further inquiries can be directed to the corresponding author.
