# Supplementary material for: Tubulin alpha-1b chain was identified as a prognosis and immune biomarker in pan-cancer combing with experimental validation in breast cancer
Source: Sci Rep. 2024 Apr 8;14:8201. doi: 10.1038/s41598-024-58982-z (PMC11001892; doi:10.1038/s41598-024-58982-z)
Supplement: Supplementary file 1 — Supplementary Information. [file 41598_2024_58982_MOESM1_ESM.pdf]

Supplementary materials for

Tubulin alpha-1b chain was identified as a prognosis and immune biomarker in pan-cancer combing with experimental validation in breast cancer

Yiyang Wang[1†], Yongxiang Li[1†], Yubo Jing[1], Yuqi Yang[2], Haiyan Wang[1], Dilimulati Ismtula[1], Chenming Guo[1*]

1 Department of Breast Surgery, Center of Digestive and Vascular, The First Affiliated Hospital of Xinjiang Medical University, Urumqi 830054, China;

2 The First Clinical Medical College of Xinjiang Medical University, Urumqi 830054, China;

*Corresponding author.

†These authors contributed equally to this work and share the first authorship.

*Correspondence: Chenming Guo, gcm_xjmu@yeah.net.

Additional material for this article can be found in the Supplementary Graphics and T able Legend module.

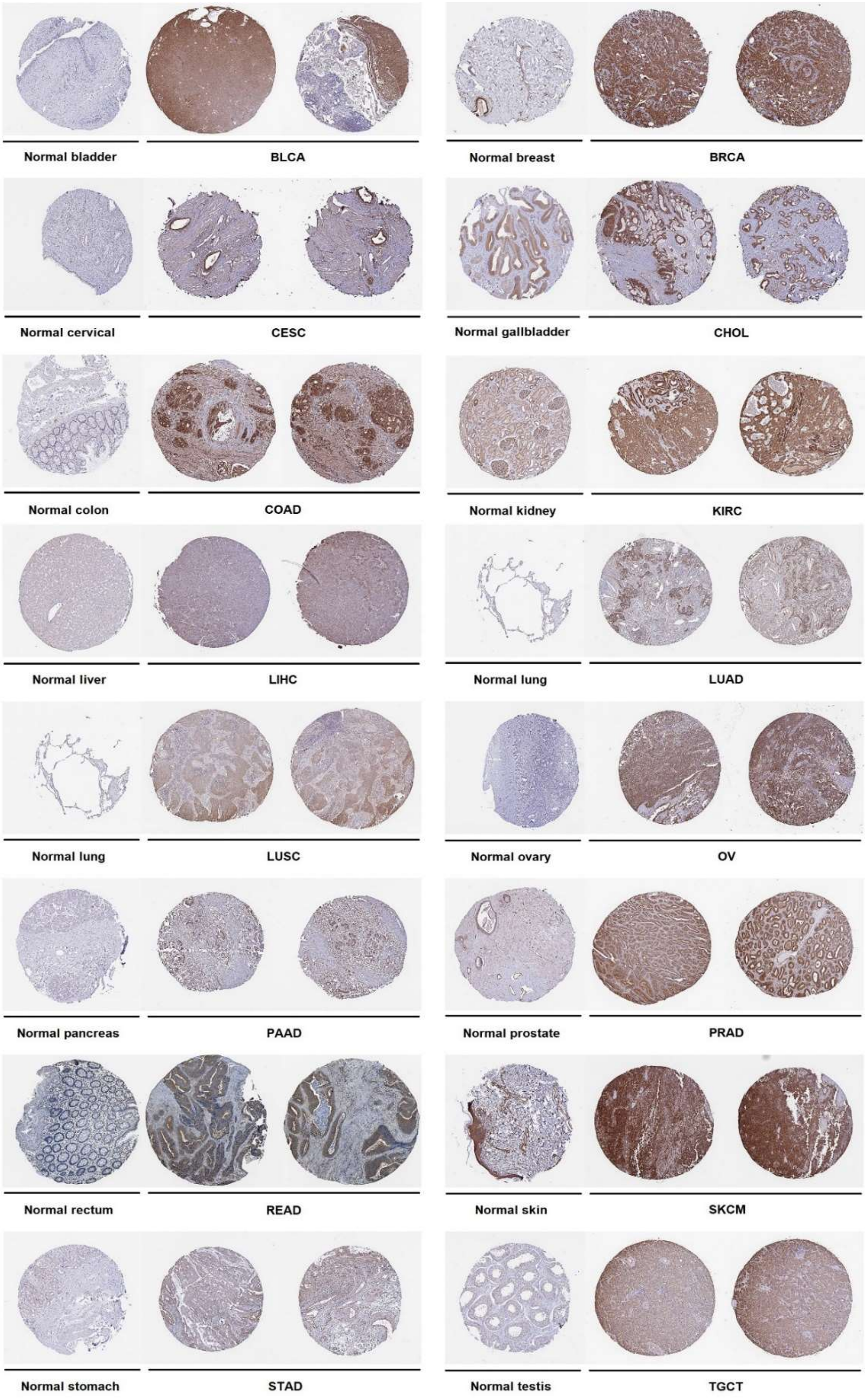

Supplementary Figure S1. Immunohistochemical Staining of 16 Normal Tissues and Tumor Tissues from the HPA Database.

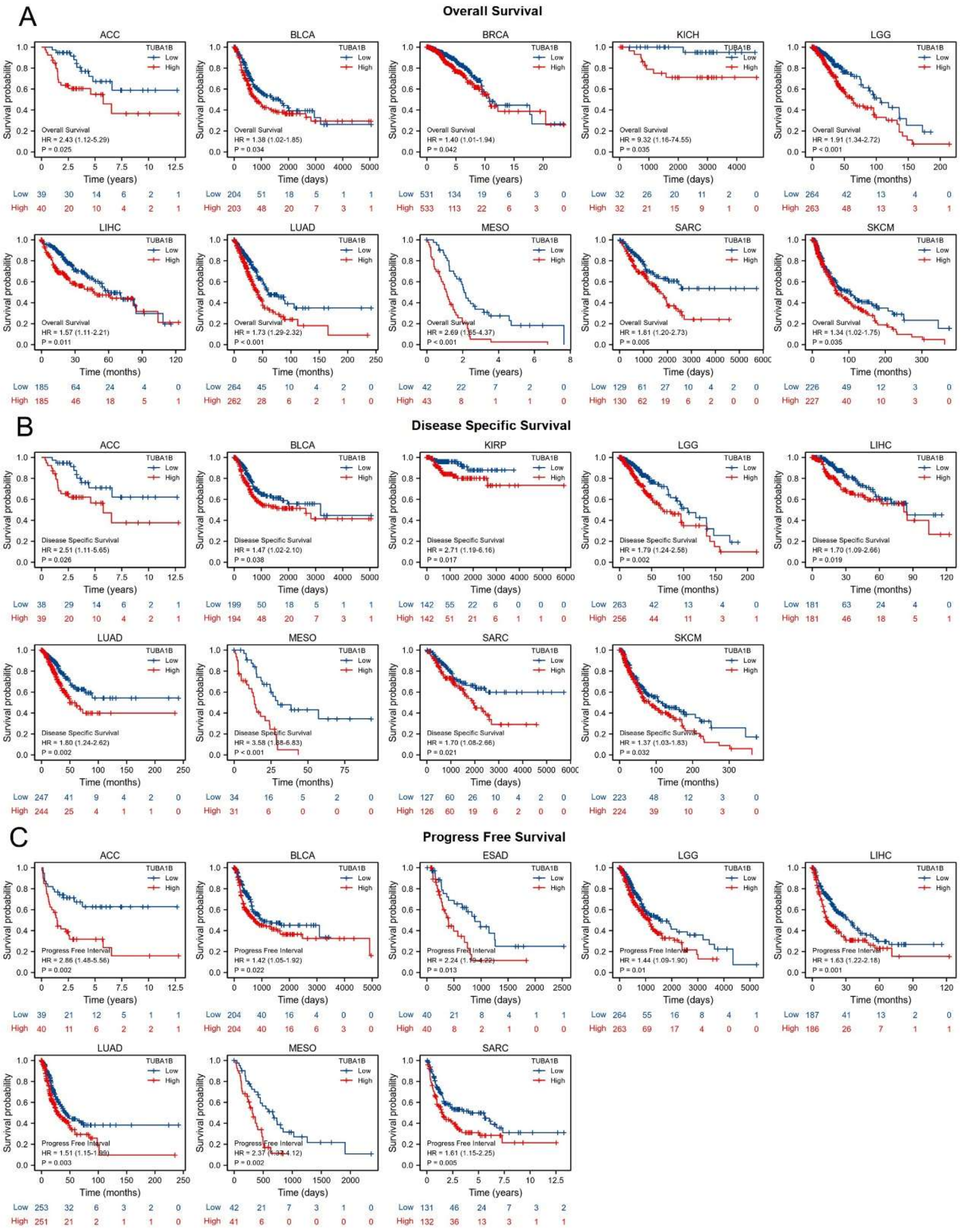

Supplementary Figure S2. Kaplan-Meier (KM) Curves Showing the Detailed Association Between TUBA1B Expression and Prognosis in Cancer Patients. **(A)** Overall Survival (OS) in ACC, BLCA, BRCA, KICH, LGG, LIHC, LUAD, MESO, SARC, and SKCM. **(B)** Disease-Specific Survival (DSS) in ACC, BLCA, KIRP, LGG, LIHC, LUAD, MESO, SARC, and SKCM. **(C)** Progression-free survival (PFS) in ACC, BLCA, E

SAD, LGG, LIHC, LUAD, MESO, and SARC.

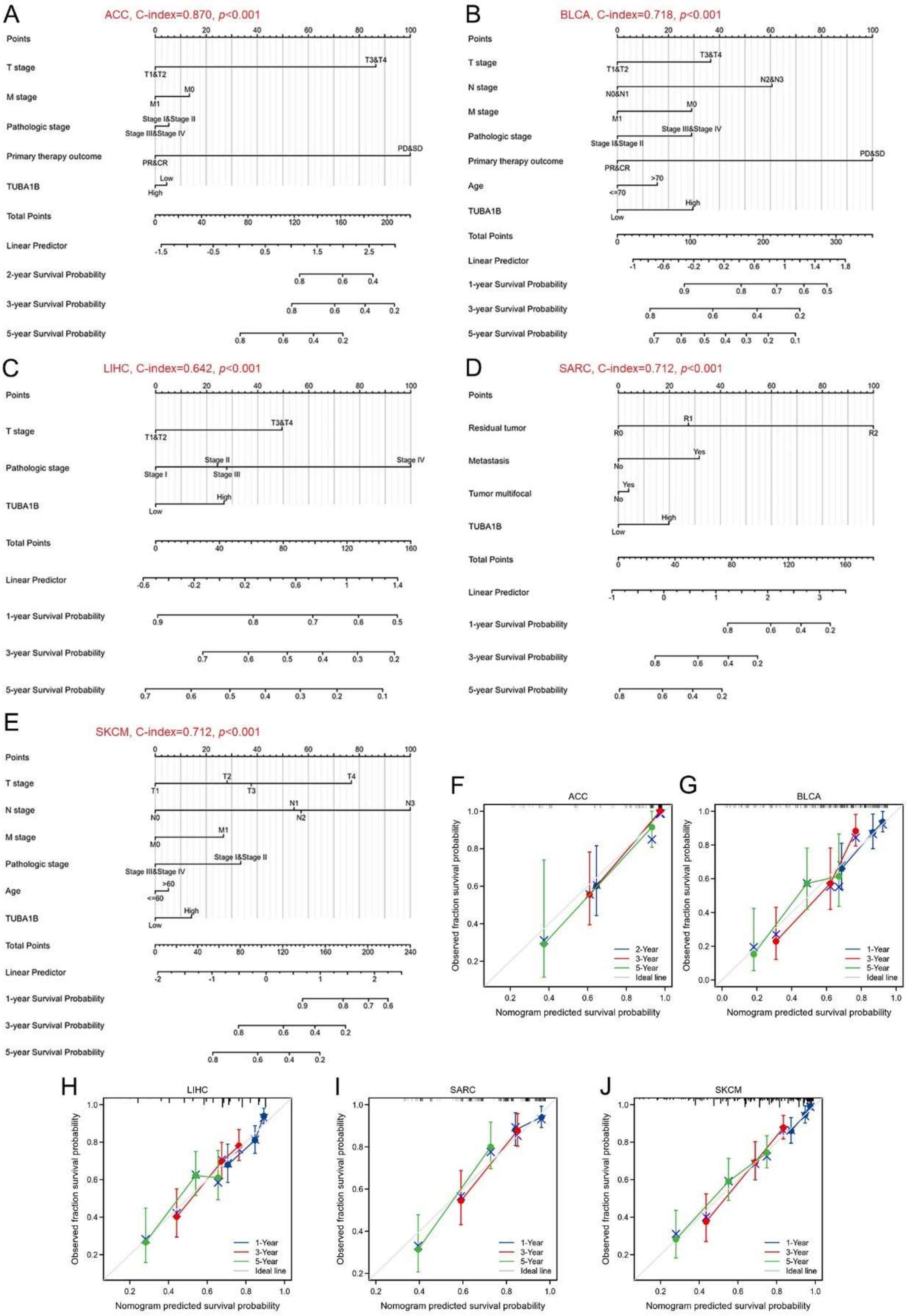

Supplementary Figure S3. Nomograms and Calibration Curves Predicting Patient OS in 5 Cancers. Nomograms of ACC **(A)**, BLCA **(B)**, LIHC **(C)**, SARC **(D)**, and SKC

M **(E)**. Calibration curves of ACC **(F)**, BLCA **(G)**, LIHC **(H)**, SARC **(I)**, SKCM **(J)**. The horizontal and vertical coordinates represent the model-predicted and actually observed survival probability, respectively. The closer each line is to the ideal line, the better the model.

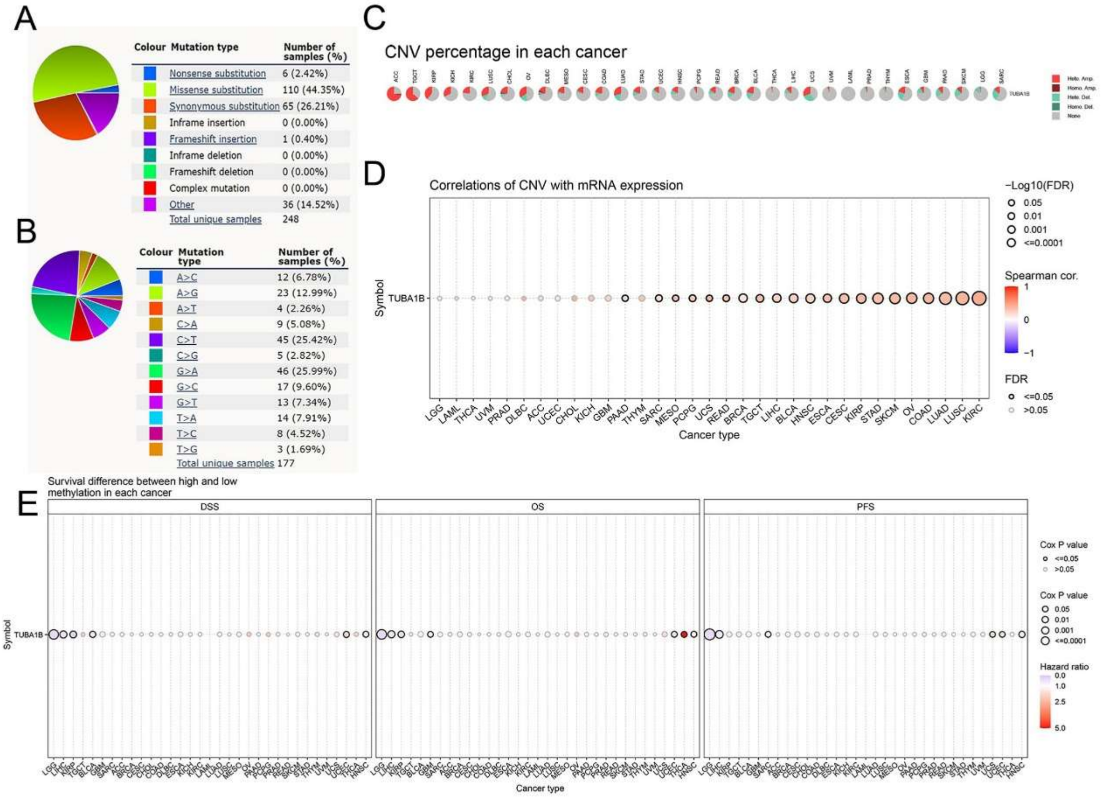

Supplementary Figure S4. Mutation analysis and epigenetic methylation analysis of TUBA1B. **(A)** The main mutation types of TUBA1B. **(B)** The main types of single nucleotide variants (SNVs) in TUBA1B. **(C)** Percentage of TUBA1B copy number variants (CNVs) in each cancer. **(D)** Correlation between TUBA1B expression and CNVs. **(E)** Effect of TUBA1B methylation levels on prognosis in cancer patients.

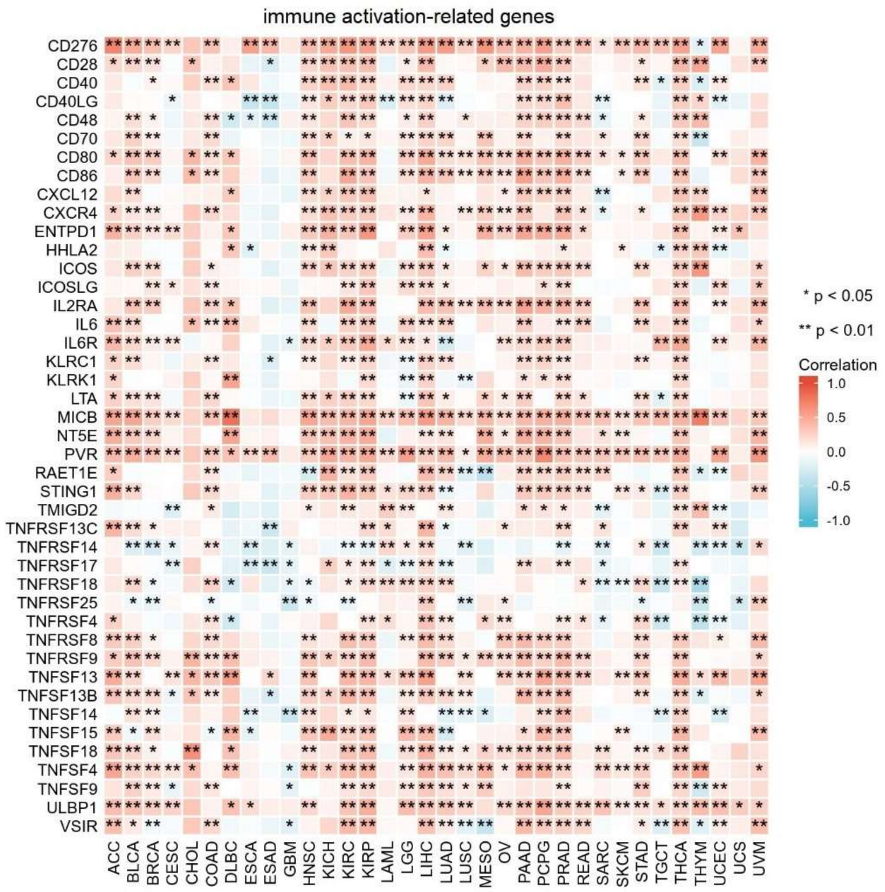

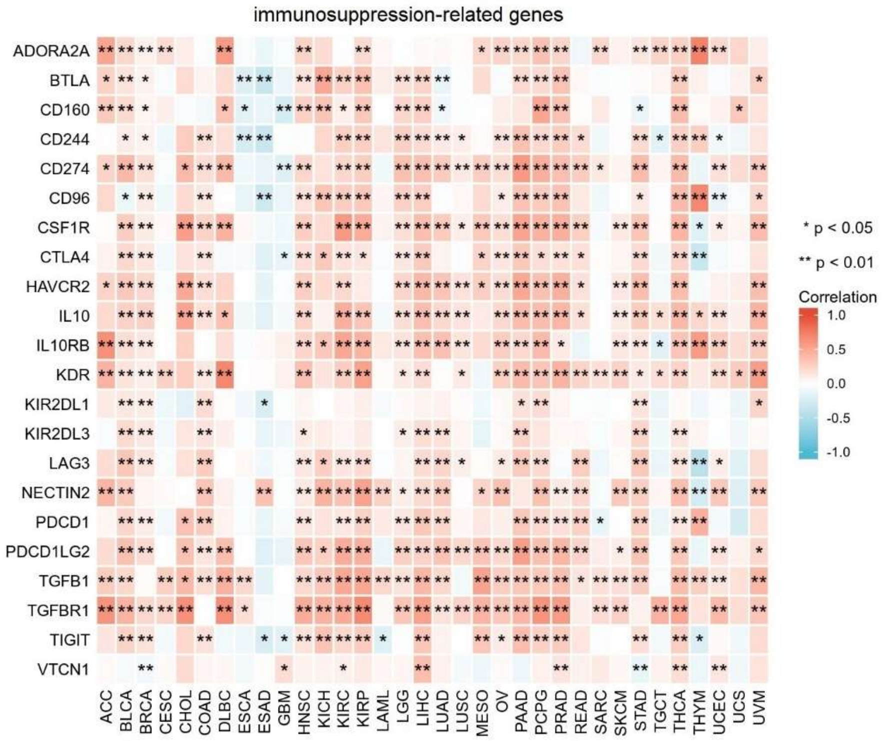

Supplementary Figure S5. Association between TUBA1B expression and immune-relat ed genes in various cancers. **(A)** Co-expression of TUBA1B with immunoactivating ge

nes. **(B)** Co-expression of TUBA1B with immunosuppressive genes. (* $p < 0.05$, ** $p < 0.01$)

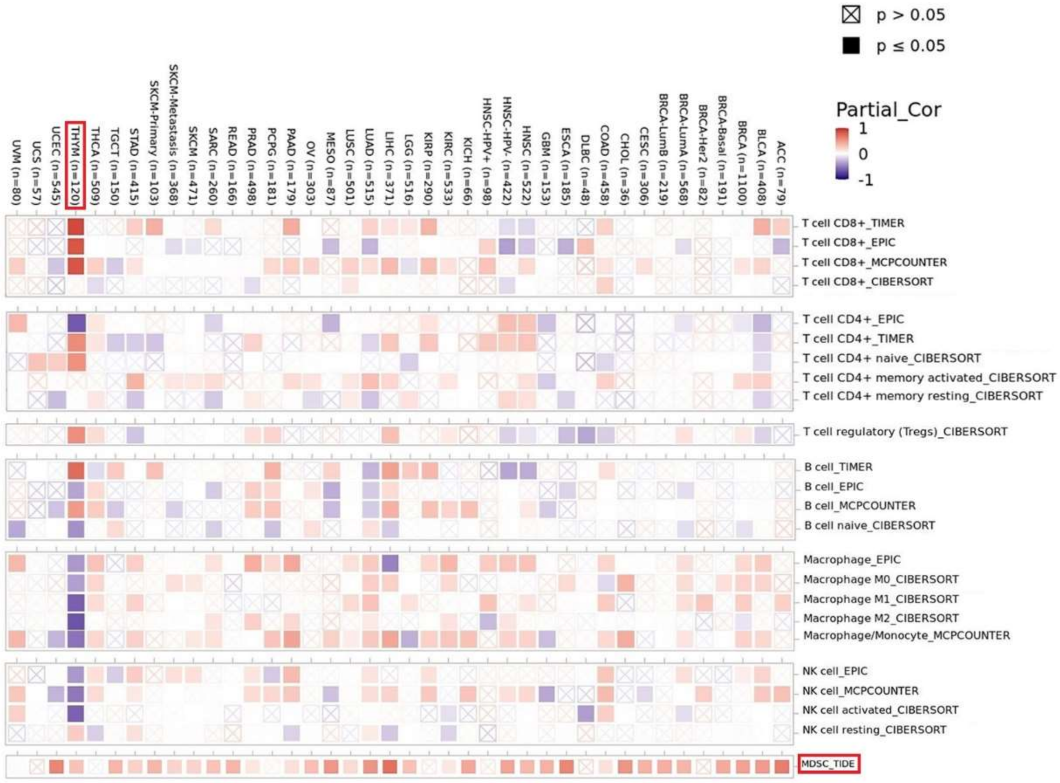

Supplementary Figure S6. Correlation analysis of TUBA1B expression with multiple immune cell infiltrates in pan-carcinogenesis.

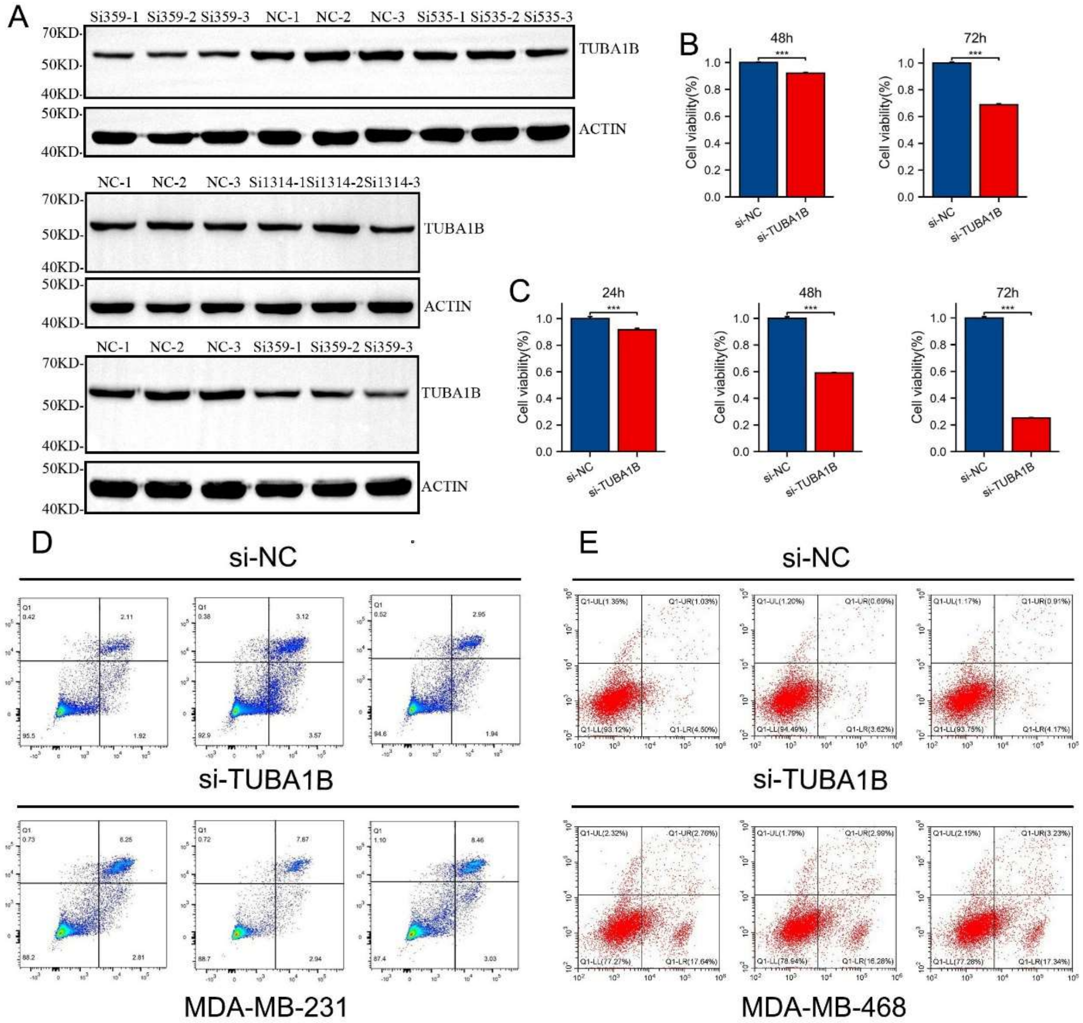

Supplementary Figure S7. Knockdown efficiency of TUBA1B and the effects on proliferation and apoptosis of breast cancer cells after TUBA1B knockdown. **(A)** Construction of TUBA1B knockdown vectors with siRNA-359, siRNA-535, and siRNA-1314. **(B-C)** Decreased viability of breast cancer cell lines after TUBA1B knockdown. **(D-E)** Flow cytometry analysis showing that knockdown of TUBA1B promoted apoptosis of breast cancer cell lines.

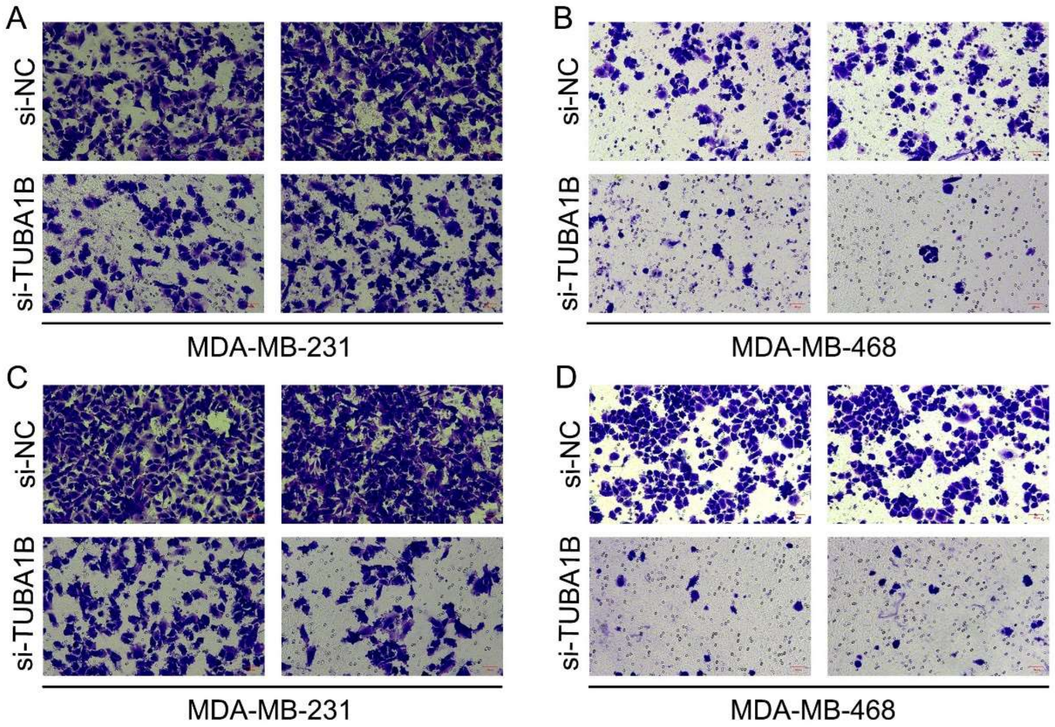

Supplementary Figure S8. Changes in the invasion and migration ability of breast ca ncer cells after TUBA1B knockdown. **(A-B)** Inhibition of invasion in breast cancer cel l lines after TUBA1B knockdown. **(C-D)** Inhibition of metastasis in breast cancer cell lines after TUBA1B knockdown. (*** $p < 0.001$)

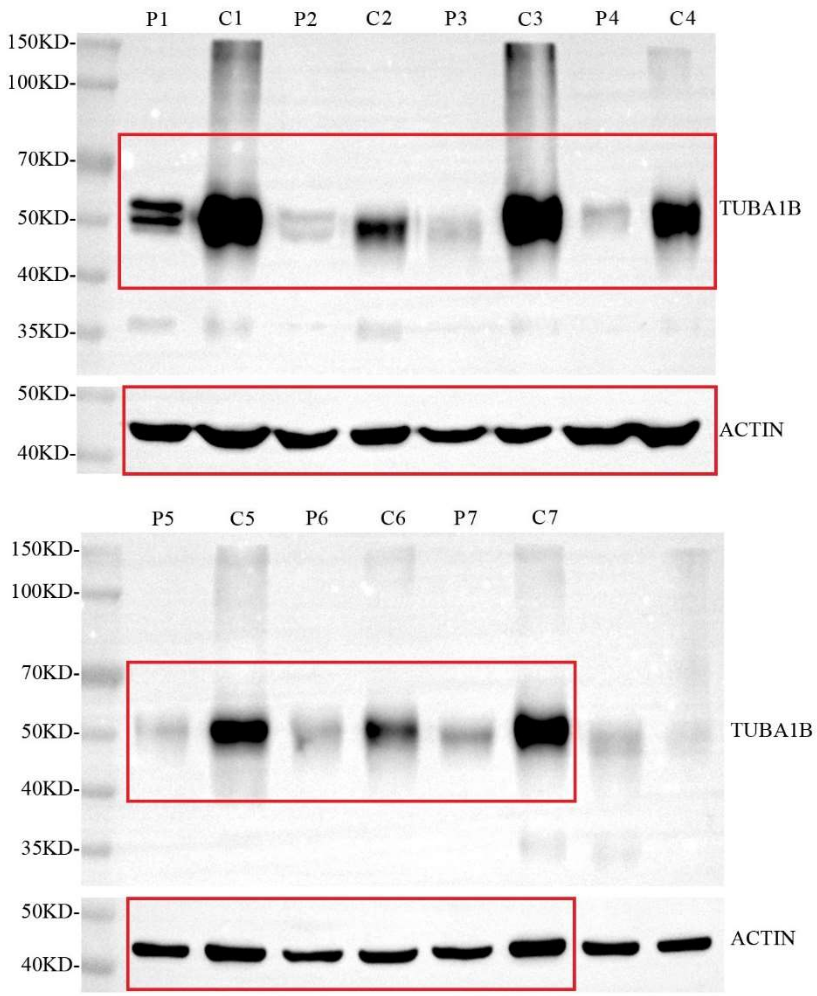

Supplementary Figure S9. Original blots of tissue validation.

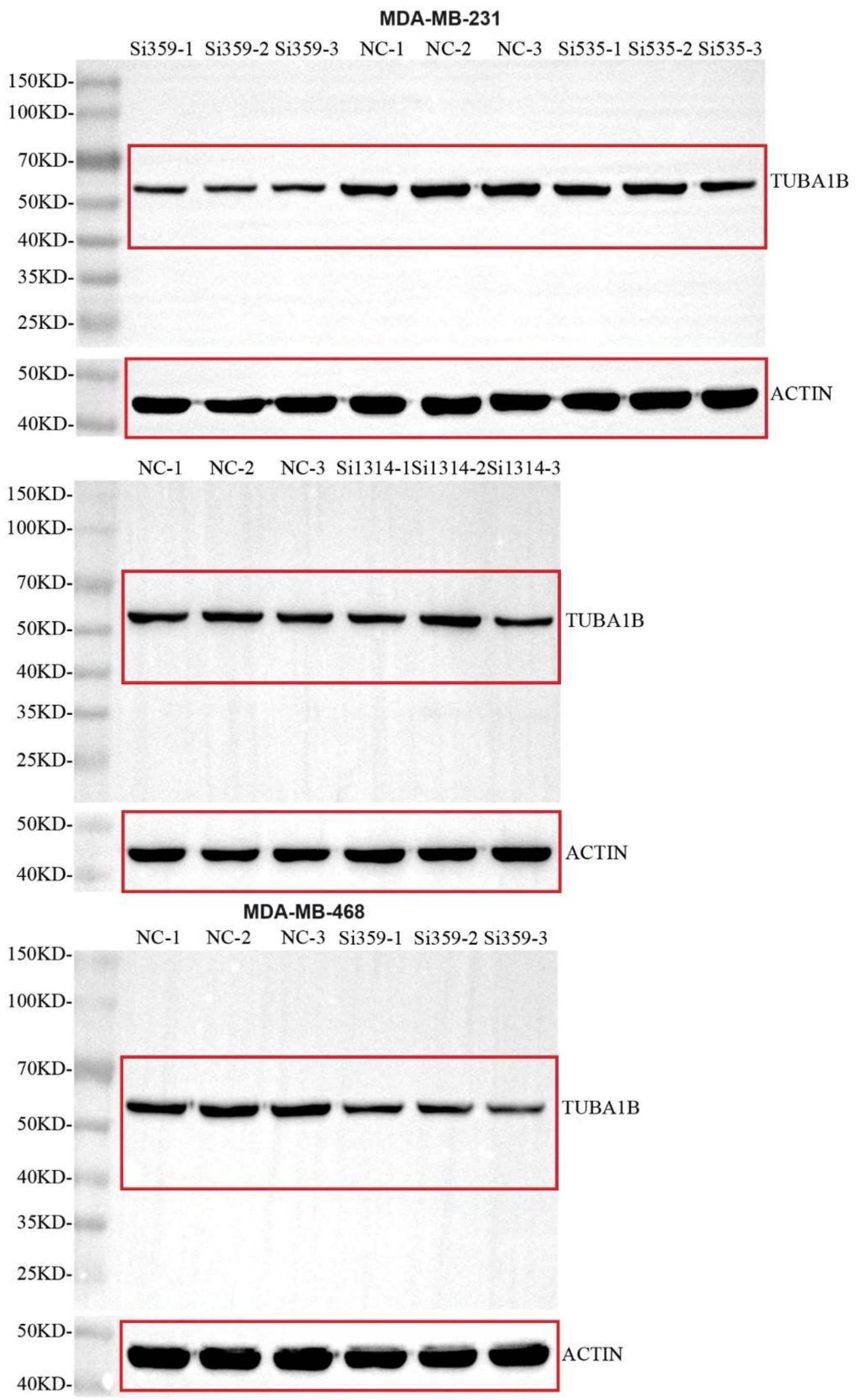

Supplementary Figure S10. Original blots of cell transfection efficiency assay.

Supplementary Table S1. Sequence information of the siRNA used.

siRNA	sense（5'-3'）	antisense（5'-3'）
si-NC	UUCUCCGAACGUGUCACGUTT	ACGUGACACGUUCGGAGAATT

si-TUBA1B-Homo-359	CCCUGAGCAGCUCAUCACATT	UGUGAUGAGCUGCUCAGGGTT
si-TUBA1B-Homo-535	CUGGGUUCACCUCCCUGCUTT	AGCAGGGAGGUGAACCCAGTT
si-TUBA1B-Homo-1314	UGGUACGUGGGUGAGGGATT	UCCCCUCACCCACGUACCATT

Supplementary Table S2. Results of univariate and multivariate Cox analysis of clinical parameters in ACC **(A)**, BLCA **(B)**, BRCA **(C)**, KICH **(D)**, LGG **(E)**, LIHC **(F)**, LUAD **(G)**, MESO **(H)**, SARC **(I)**, and SKCM **(J)**.

A. ACC

Characteristics	Total(N)	Univariate analysis		Multivariate analysis	
		Hazard ratio (95% CI)	P value	Hazard ratio (95% CI)	P value
T stage	77				
T1&T2	51	Reference			
T3&T4	26	10.286 (3.976-26.608)	**<0.001**	6.886 (0.845-56.092)	0.071
N stage	77				
N0	68	Reference			
N1	9	2.038 (0.769-5.400)	0.152		
M stage	77				
M0	62	Reference			
M1	15	6.150 (2.710-13.959)	**<0.001**	0.742 (0.205-2.678)	0.648
Pathologic stage	77				

Characteristics	Total(N)	Univariate analysis		Multivariate analysis	
		Hazard ratio (95% CI)	P value	Hazard ratio (95% CI)	P value
Stage I&Stage II	46	Reference			
Stage III&Stage IV	31	6.476 (2.706-15.498)	**<0.001**	0.888 (0.085-9.230)	0.921
Radiation therapy	76				
No	59	Reference			
Yes	17	1.292 (0.512-3.262)	0.587		
Primary therapy outcome	67				
PD&SD	20	Reference			
PR&CR	47	0.063 (0.020-0.199)	**<0.001**	0.108 (0.028-0.420)	**0.001**
Gender	79				
Female	48	Reference			
Male	31	1.001 (0.469-2.137)	0.999		
Age	79				
<=50	41	Reference			
>50	38	1.799 (0.846-3.824)	0.127		
TUBA1B	79				
Low	39	Reference			

Characteristics	Total(N)	Univariate analysis		Multivariate analysis	
		Hazard ratio (95% CI)	P value	Hazard ratio (95% CI)	P value
High	40	2.435 (1.120-5.293)	**0.025**	0.905 (0.315-2.601)	0.852

B. BLCA

Characteristics	Total(N)	Univariate analysis		Multivariate analysis	
		Hazard ratio (95% CI)	P value	Hazard ratio (95% CI)	P value
T stage	379				
T1&T2	124	Reference			
T3&T4	255	2.199 (1.515-3.193)	**<0.001**	1.422 (0.428-4.726)	0.566
N stage	369				
N0&N1	285	Reference			
N2&N3	84	2.273 (1.640-3.150)	**<0.001**	1.791 (0.922-3.477)	0.085
M stage	213				
M0	202	Reference			
M1	11	3.136 (1.503-6.544)	**0.002**	0.756 (0.238-2.401)	0.635
Pathologic stage	411				
Stage I&Stage II	134	Reference			

Characteristics	Total(N)	Univariate analysis		Multivariate analysis	
		Hazard ratio (95% CI)	P value	Hazard ratio (95% CI)	P value
Stage III&Stage IV	277	2.310 (1.596-3.342)	**<0.001**	1.323 (0.383-4.572)	0.658
Primary therapy outcome	357				
PD&SD	101	Reference			
PR&CR	256	0.226 (0.162-0.315)	**<0.001**	0.382 (0.212-0.688)	**0.001**
Age	413				
<=70	233	Reference			
>70	180	1.421 (1.063-1.901)	**0.018**	1.162 (0.696-1.940)	0.565
Radiation therapy	387				
No	366	Reference			
Yes	21	0.965 (0.475-1.964)	0.923		
Gender	413				
Female	109	Reference			
Male	304	0.849 (0.616-1.169)	0.316		
TUBA1B	413				
Low	207	Reference			
High	206	1.359 (1.013-1.822)	**0.041**	1.329 (0.786-2.245)	0.288

C. BRCA

Characteristics	Total(N)	Univariate analysis		Multivariate analysis	
		Hazard ratio (95% CI)	P value	Hazard ratio (95% CI)	P value
T stage	1079				
T1&T2	905	Reference			
T3&T4	174	1.608 (1.110-2.329)	**0.012**	0.960 (0.537-1.717)	0.891
N stage	1063				
N0	514	Reference			
N1&N2&N3	549	2.239 (1.567-3.199)	**<0.001**	1.418 (0.800-2.512)	0.232
M stage	922				
M0	902	Reference			
M1	20	4.254 (2.468-7.334)	**<0.001**	1.716 (0.758-3.884)	0.195
Pathologic stage	1059				
Stage I&Stage II	799	Reference			
Stage III&Stage IV	260	2.391 (1.703-3.355)	**<0.001**	3.048 (1.525-6.092)	**0.002**
Age	1082				
<=60	601	Reference			
>60	481	2.020 (1.465-2.784)	**<0.001**	2.391 (1.521-3.761)	**<0.001**

Characteristics	Total(N)	Univariate analysis		Multivariate analysis	
		Hazard ratio (95% CI)	P value	Hazard ratio (95% CI)	P value
radiation_therapy	986				
No	434	Reference			
Yes	552	0.576 (0.394-0.841)	**0.004**	0.427 (0.267-0.683)	**<0.001**
TUBA1B	1082				
Low	540	Reference			
High	542	1.329 (0.965-1.829)	0.082	1.808 (1.158-2.825)	**0.009**

D. KICH

Characteristics	Total(N)	Univariate analysis		Multivariate analysis	
		Hazard ratio (95% CI)	P value	Hazard ratio (95% CI)	P value
T stage	64				
T1&T2	44	Reference			
T3&T4	20	10.121 (2.098-48.814)	**0.004**	11.318 (2.239-57.208)	**0.003**
Gender	64				
Female	26	Reference			
Male	38	1.528 (0.381-6.125)	0.550		
Age	64				

Characteristics	Total(N)	Univariate analysis		Multivariate analysis	
		Hazard ratio (95% CI)	P value	Hazard ratio (95% CI)	P value
<=50	33	Reference			
>50	31	3.847 (0.797-18.561)	0.093	2.374 (0.477-11.822)	0.291
TUBA1B	64				
Low	32	Reference			
High	32	9.315 (1.164-74.548)	**0.035**	11.480 (1.397-94.329)	**0.023**

E. LGG

Characteristics	Total(N)	Univariate analysis		Multivariate analysis	
		Hazard ratio (95% CI)	P value	Hazard ratio (95% CI)	P value
WHO grade	466				
G2	223	Reference			
G3	243	3.059 (2.046-4.573)	**<0.001**	2.696 (1.745-4.165)	**<0.001**
Primary therapy outcome	457				
PD&SD	256	Reference			
PR&CR	201	0.202 (0.113-0.359)	**<0.001**	0.243 (0.129-0.458)	**<0.001**
Age	527				
<=40	264	Reference			

Characteristics	Total(N)	Univariate analysis		Multivariate analysis	
		Hazard ratio (95% CI)	P value	Hazard ratio (95% CI)	P value
>40	263	2.889 (2.009-4.155)	**<0.001**	2.902 (1.884-4.470)	**<0.001**
Gender	527				
Female	238	Reference			
Male	289	1.124 (0.800-1.580)	0.499		
TUBA1B	527				
Low	264	Reference			
High	263	1.907 (1.335-2.723)	**<0.001**	1.653 (1.076-2.538)	**0.022**

F. LIHC

Characteristics	Total(N)	Univariate analysis		Multivariate analysis	
		Hazard ratio (95% CI)	P value	Hazard ratio (95% CI)	P value
T stage	370				
T1&T2	277	Reference			
T3&T4	93	2.598 (1.826-3.697)	**<0.001**	1.861 (0.254-13.626)	0.541
N stage	258				
N0	254	Reference			
N1	4	2.029 (0.497-8.281)	0.324		

Characteristics	Total(N)	Univariate analysis		Multivariate analysis	
		Hazard ratio (95% CI)	P value	Hazard ratio (95% CI)	P value
Pathologic stage	349				
Stage I	173	Reference			
Stage II	86	1.417 (0.868-2.312)	0.164	1.355 (0.828-2.216)	0.227
Stage III	85	2.734 (1.792-4.172)	**<0.001**	1.418 (0.192-10.442)	0.732
Stage IV	5	5.597 (1.726-18.148)	**0.004**	3.489 (0.351-34.731)	0.286
Gender	373				
Female	121	Reference			
Male	252	0.793 (0.557-1.130)	0.200		
Age	373				
<=60	177	Reference			
>60	196	1.205 (0.850-1.708)	0.295		
TUBA1B	373				
Low	187	Reference			
High	186	1.479 (1.047-2.090)	**0.027**	1.399 (0.958-2.042)	0.082

G. LUAD

Characteristics	Total(N)	Univariate analysis		Multivariate analysis	
		Hazard ratio (95% CI)	P value	Hazard ratio (95% CI)	P value
T stage	523				
T1	175	Reference			
T2	282	1.521 (1.068-2.166)	**0.020**	1.417 (0.865-2.321)	0.166
T3&T4	66	3.066 (1.950-4.823)	**<0.001**	1.922 (0.947-3.902)	0.070
N stage	510				
N0	343	Reference			
N1&N2&N3	167	2.601 (1.944-3.480)	**<0.001**	1.602 (1.014-2.533)	**0.044**
M stage	377				
M0	352	Reference			
M1	25	2.136 (1.248-3.653)	**0.006**	1.248 (0.517-3.013)	0.622
Pathologic stage	518				
Stage I&Stage II	411	Reference			
Stage III&Stage IV	107	2.664 (1.960-3.621)	**<0.001**	1.470 (0.814-2.654)	0.201
Gender	526				
Female	280	Reference			
Male	246	1.070 (0.803-1.426)	0.642		

Characteristics	Total(N)	Univariate analysis		Multivariate analysis	
		Hazard ratio (95% CI)	P value	Hazard ratio (95% CI)	P value
Age	516				
<=65	255	Reference			
>65	261	1.223 (0.916-1.635)	0.172		
Primary therapy outcome	439				
PD&SD	108	Reference			
PR&CR	331	0.377 (0.268-0.530)	**<0.001**	0.376 (0.249-0.567)	**<0.001**
TUBA1B	526				
Low	264	Reference			
High	262	1.732 (1.293-2.320)	**<0.001**	1.576 (1.041-2.385)	**0.031**

H. MESO

Characteristics	Total(N)	Univariate analysis		Multivariate analysis	
		Hazard ratio (95% CI)	P value	Hazard ratio (95% CI)	P value
T stage	83				
T1&T2	39	Reference			
T3&T4	44	0.955 (0.590-1.547)	0.852		
N stage	81				

Characteristics	Total(N)	Univariate analysis		Multivariate analysis	
		Hazard ratio (95% CI)	P value	Hazard ratio (95% CI)	P value
N0	43	Reference			
N1&N2&N3	38	0.904 (0.557-1.467)	0.683		
M stage	59				
M0	56	Reference			
M1	3	1.917 (0.454-8.089)	0.376		
Pathologic stage	85				
Stage I&Stage II	26	Reference			
Stage III&Stage IV	59	0.974 (0.577-1.647)	0.923		
Radiation therapy	84				
No	59	Reference			
Yes	25	0.693 (0.409-1.176)	0.174		
Gender	85				
Female	15	Reference			
Male	70	0.944 (0.516-1.726)	0.850		
Age	85				
<=65	46	Reference			

Characteristics	Total(N)	Univariate analysis		Multivariate analysis	
		Hazard ratio (95% CI)	P value	Hazard ratio (95% CI)	P value
>65	39	1.296 (0.805-2.085)	0.286		
TUBA1B	85				
Low	42	Reference			
High	43	2.689 (1.653-4.373)	**<0.001**	2.689 (1.653-4.373)	**<0.001**

I. SARC

Characteristics	Total(N)	Univariate analysis		Multivariate analysis	
		Hazard ratio (95% CI)	P value	Hazard ratio (95% CI)	P value
Radiation ther apy	257				
No	179	Reference			
Yes	78	0.864 (0.557-1.339)	0.513		
Gender	263				
Female	144	Reference			
Male	119	0.905 (0.607-1.349)	0.623		
Age	263				
<=60	130	Reference			
>60	133	1.285 (0.864-1.911)	0.216		

Characteristics	Total(N)	Univariate analysis		Multivariate analysis	
		Hazard ratio (95% CI)	P value	Hazard ratio (95% CI)	P value
Residual tumor	235				
R0	157	Reference			
R1	69	2.255 (1.437-3.538)	**<0.001**	2.115 (1.142-3.916)	**0.017**
R2	9	11.328 (5.225-24.561)	**<0.001**	15.315 (5.170-45.368)	**<0.001**
Metastasis	179				
No	120	Reference			
Yes	59	2.888 (1.762-4.732)	**<0.001**	2.377 (1.360-4.155)	**0.002**
Tumor multifocal	239				
No	199	Reference			
Yes	40	2.402 (1.502-3.840)	**<0.001**	1.116 (0.484-2.574)	0.797
TUBA1B	263				
Low	131	Reference			
High	132	1.764 (1.173-2.652)	**0.006**	1.716 (0.965-3.052)	0.066

J. SKCM

Characteristics	Total(N)	Univariate analysis		Multivariate analysis	
		Hazard ratio (95% CI)	P value	Hazard ratio (95% CI)	P value
T stage	361				
T1	41	Reference			
T2	77	1.495 (0.811-2.756)	0.197	1.739 (0.882-3.426)	0.110
T3	90	2.097 (1.158-3.798)	**0.015**	2.094 (1.068-4.103)	**0.031**
T4	153	3.711 (2.070-6.653)	**<0.001**	4.522 (2.307-8.866)	**<0.001**
N stage	402				
N0	224	Reference			
N1	73	1.497 (1.014-2.210)	**0.043**	2.911 (0.997-8.496)	0.051
N2	49	1.534 (0.972-2.419)	0.066	3.071 (1.018-9.260)	**0.046**
N3	56	2.731 (1.769-4.215)	**<0.001**	7.113 (2.355-21.480)	**<0.001**
M stage	430				
M0	406	Reference			
M1	24	1.897 (1.029-3.496)	**0.040**	1.688 (0.690-4.132)	0.252
Pathologic stage	410				
Stage I&Stage II	217	Reference			
Stage III&Stage IV	193	1.617 (1.207-2.165)	**0.001**	0.518 (0.183-1.464)	0.215

Characteristics	Total(N)	Univariate analysis		Multivariate analysis	
		Hazard ratio (95% CI)	P value	Hazard ratio (95% CI)	P value
Radiation therapy	450				
No	374	Reference			
Yes	76	0.977 (0.694-1.377)	0.895		
Gender	456				
Female	172	Reference			
Male	284	1.172 (0.879-1.563)	0.281		
Age	456				
<=60	246	Reference			
>60	210	1.656 (1.251-2.192)	**<0.001**	1.107 (0.790-1.551)	0.555
TUBA1B	456				
Low	227	Reference			
High	229	1.329 (1.015-1.740)	**0.039**	1.321 (0.955-1.828)	0.093

Supplementary Table S3. List of the top 100 genes associated with TUBA1B.

Supplementary Table S4. GO terms and KEGG pathways enriched in the analysis.
